# Rational Design of a Multifunctional MOFs for Alkane‐Selective Gas Separation

**DOI:** 10.1002/advs.202516118

**Published:** 2025-10-14

**Authors:** Li Wang, Zhaozhuang Liu, Yating Wang, Jiaqi Liu, Jinping Li, Jiangfeng Yang

**Affiliations:** ^1^ College of Chemical Engineering and Technology Taiyuan University of Technology Taiyuan Shanxi 030024 China; ^2^ Shanxi Research Institute of Huairou Laboratory Taiyuan Shanxi 030024 China

**Keywords:** alkane‐selective, MOFs, multifunctional adsorbents, separation

## Abstract

Efficient separation of light hydrocarbons—including the removal of CH_4_ from N_2_ and the purification of olefins such as C_2_H_4_ and C_3_H_6_ from their corresponding alkanes (C_2_H_6_ and C_3_H_8_)—is critical in natural gas upgrading, steam cracking, and downstream petrochemical production. Traditional adsorbents are tailored to specific mixtures, limiting their broader applicability. The development of multifunctional adsorbents that can efficiently operate across multiple gas separation systems represents a promising strategy to simplify material design and broaden industrial relevance. Herein, methyl‐functional groups are innovatively introduced into porous coordination polymers (PCPs), synthesizing PCP‐BDC‐M and PCP‐BDC‐DM with precisely tailored microporous structures. Notably, the dimethyl‐functionalized PCP‐BDC‐DM demonstrates superior multifunctional selectivity toward CH_4_/N_2_, C_2_H_6_/C_2_H_4_, and C_3_H_8_/C_3_H_6_ gas mixtures. Adsorption isotherms and Ideal Adsorbed Solution Theory (IAST) calculations reveal significantly higher alkane selectivity in PCP‐BDC‐DM compared to PCP‐BDC‐M and existing alkane‐selective adsorbents. Grand Canonical Monte Carlo (GCMC) simulations provide molecular‐level insight, confirming that methyl groups effectively enhance interactions between alkane molecules and the framework. Dynamic breakthrough experiments further validate PCP‐BDC‐DM's excellent practical separation capability and structural stability. This study offers valuable insights into designing advanced adsorbents for alkane‐selective gas separation.

## Graphical abstract

The design of a dimethyl‐functionalized adsorbent provides insight into the simultaneous separation of CH_4_/N_2_, C_2_H_6_/C_2_H_4,_ and C_3_H_8_/C_3_H_6_ mixtures.

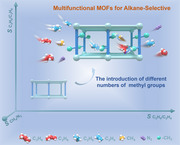

## 1 Introduction

The efficient separation and purification of chemical mixtures, particularly involving light hydrocarbons, remain energy‐intensive and economically challenging processes in the chemical industry.^[^ ^1^ ^]^ Adsorptive separation techniques based on porous materials have emerged as low‐carbon alternatives that could potentially replace traditional thermally driven distillation methods.^[^ ^2^ ^]^ Among these, the separations involving methane/nitrogen (CH_4_/N_2_) and olefins (C_2_H_4_ and C_3_H_6_) from their corresponding alkanes (C_2_H_6_ and C_3_H_8_) represent crucial tasks across multiple sectors, including natural gas processing, olefin production, and downstream petrochemical manufacturing.^[^ ^3^, ^4^, ^5^ ^]^

CH_4_, the main component of natural gas, is a clean energy carrier with a high heating value and is widely used.^[^ ^6^, ^7^ ^]^ Its consumption continues to grow, surpassing that of conventional fossil fuels.^[^ ^8^ ^]^ In response to the growing demand, coalbed methane (CBM), a typical form of unconventional natural gas, has been increasingly utilized as a supplementary source. However, CBM is often diluted with air during extraction, undermine, lowering CH_4_ concentration, and limiting its direct use. Consequently, most of the low concentration CBM was vented to the air, exacerbating the greenhouse effect.^[^ ^9^ ^]^ Given that nitrogen is the primary impurity, developing alkane‐selective adsorbents for efficient CH_4_/N_2_ separation is crucial for its utilization. Olefins (C_2_H_4_ and C_3_H_6_), often polymer‐grade or even ultra‐high purity (99.99%), are required to produce advanced fine chemicals and polymers.^[^ ^10^, ^11^ ^]^ However, in industrial olefin production processes, such as steam cracking, the desired products are typically produced alongside alkanes. Therefore, effective separation strategies are critical to isolate high‐purity olefins.^[^ ^12^ ^]^ According to the reports, alkane‐selective adsorption process could obtain olefin directly through a single adsorption step, significantly simplifying the separation process, so having superior advantages over olefin‐selective adsorbents.^[^ ^13^, ^14^, ^15^, ^16^, ^17^, ^18^ ^]^

A variety of alkane‐selective adsorbents have been reported to fulfill the above separation scenarios.^[^ ^19^, ^20^, ^21^, ^22^, ^23^, ^24^, ^25^, ^26^ ^]^ Nevertheless, most reported adsorbents exhibit selectivity toward only one particular gas pair, which limits their versatility and necessitates case‐by‐case material development. In contrast, multifunctional alkane‐selective adsorbents provide broader applicability using a single material, which reduces material complexity and improves adaptability in changing industrial conditions. Despite these potential benefits, multifunctional alkane‐selective adsorbents remain rare, primarily due to the difficulty of achieving selectivity across gas pairs with different molecular sizes and polarizabilities.^[^ ^27^, ^28^ ^]^ Therefore, the rational design of porous materials that can simultaneously exhibit high selectivity for CH_4_/N_2_, C_2_H_6_/C_2_H_4_, and C_3_H_8_/C_3_H_6_ represents a valuable yet challenging task.

To address this challenge, we designed and synthesized a series of methyl‐functionalized porous coordination polymers (PCPs) with the goal of achieving multifunctional alkane‐selective adsorption.^[^ ^29^, ^30^ ^]^ Specifically, we constructed two isoreticular frameworks, [Co(bdc‐m)(dpg)]n (PCP‐BDC‐M; bdc‐m = 2‐Methyl‐1,4‐benzenedicarboxylic acid, dpg = meso‐a,b‐di(4‐pyridyl) glycol) and [Co(bdc‐dm)(dpg)]n (PCP‐BDC‐DM; bdc‐dm = 2,5‐dimethylterephthalic acid), by incorporating mono‐ and di‐methyl‐substituted terephthalic acid linkers into a 1D channel architecture. The introduction of methyl groups was intended to enhance framework–alkane interactions, thereby promoting alkane recognition across different gas pairs. And these PCPs exhibit remarkable separation performance in three representative hydrocarbon systems—CH_4_/N_2_, C_2_H_6_/C_2_H_4_, and C_3_H_8_/C_3_H_6_—demonstrating their potential as versatile, multifunctional adsorbents. Grand Canonical Monte Carlo (GCMC) simulations and dynamic breakthrough experiments were further employed to elucidate the adsorption mechanisms and validate practical separation capability. This work highlights a rational design strategy for constructing a single‐adsorbent capable of addressing multiple industrially relevant separations.

## 2 Results and Discussion

**Figure 1:**
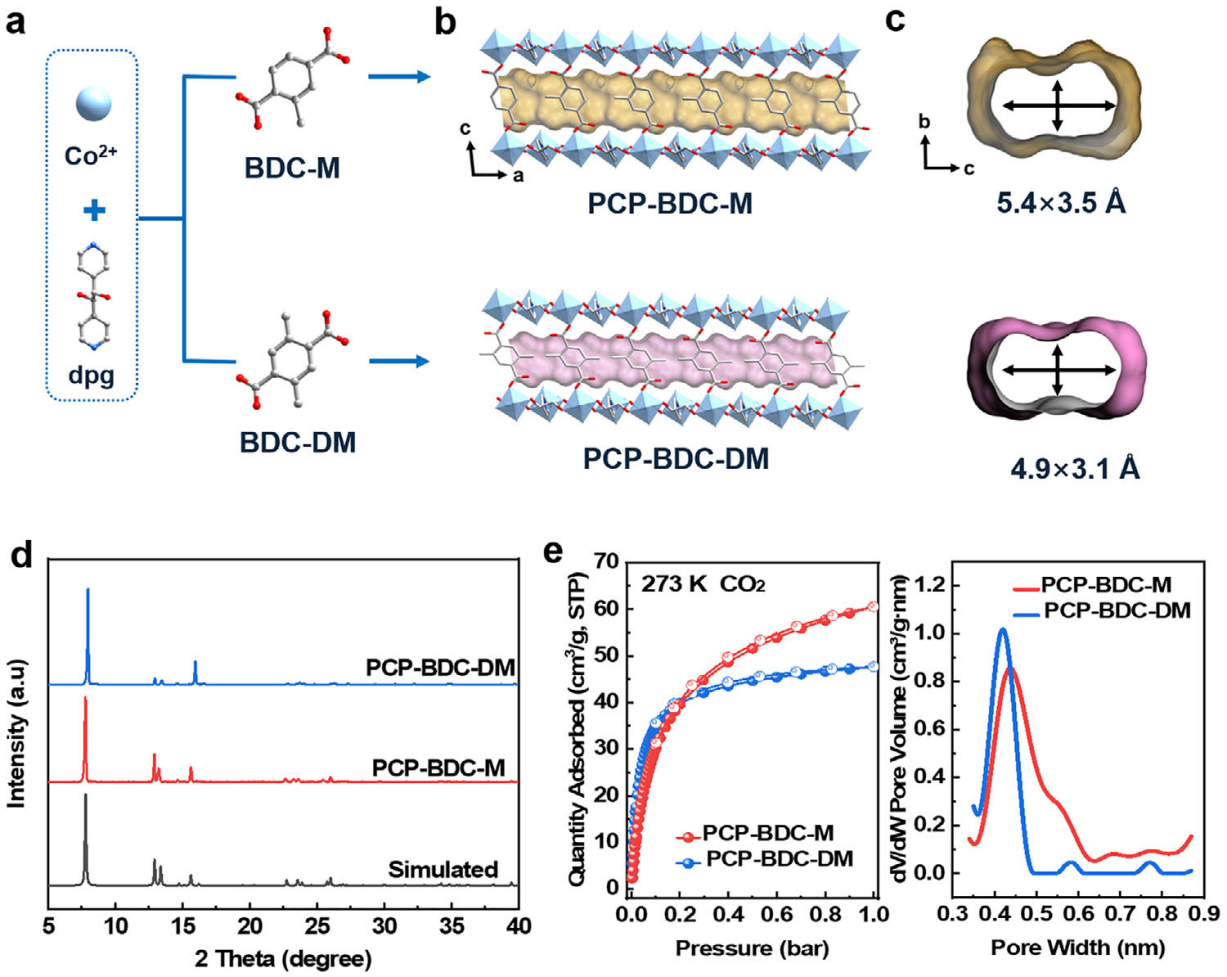
Structure scheme of PCP‐BDC‐M and PCP‐BDC‐DM a), PXRD patterns b) and CO_2_ sorption isotherms at 273 K and pore size distribution c).

Methyl‐functionalized PCP‐BDC‐M and PCP‐BDC‐DM were successfully synthesized (Figure 1d). Structural confirmation was achieved through Rietveld refinement and preliminary verification via Le Bail fitting of powder X‐ray diffraction data (Figures S1 and S2 and Table S1, Supporting Information). The results indicate that PCP‐BDC‐M and PCP‐BDC‐DM are phase‐pure, with their structures shown in Figures S3 and S4 (Supporting Information) and 1d. In both structures, the Co center adopts an octahedral coordination geometry, being coordinated by two nitrogen atoms from the pyridine rings, two oxygen atoms from the hydroxyl groups of the dpg ligands, and two oxygen atoms from the carboxyl groups of the methyl‐substituted terephthalic acid ligands. This arrangement forms 1D straight channels with pore sizes of ≈5.4 × 3.5 and 4.9 × 3.1 Å, respectively (**Figure** **1**a–c). Thermogravimetric analysis revealed that all solvent‐exchange samples can be fully desolvated at 393 K and exhibit high thermal stability (Figure S5, Supporting Information). N_2_ adsorption isotherms at 77 K were measured, as the number of methyl groups increases, the adsorption capacity of N_2_ gradually decreases, and the N_2_ adsorption amount on PCP‐BDC‐DM is basically zero. (Figure S6, Supporting Information). In this way, CO_2_ adsorption isotherms at 273 K were used to investigate the specific surface areas and porosities of the materials. As shown in Figure 1e, the Brunauer‐Emmett‐Teller specific surface areas of PCP‐BDC‐M and PCP‐BDC‐DM were calculated to be 248.18 and 186.49 m^2^ g^−1^, respectively, indicating a gradual decrease associated with the increased number of methyl groups. Furthermore, the pore‐size distributions, determined by the Horvath–Kawazoe method, revealed pore sizes of ≈4.4 Å for PCP‐BDC‐M and 4.2 Å for PCP‐BDC‐DM (Table S2, Supporting Information). This observation indicates that additional methyl substitution slightly reduces the effective pore dimensions. Contact angle test and water adsorption isotherms indicate the hydrophobic property of PCP‐BDC‐DM is excellent, providing a foundation for its subsequent industrial application. (Figure S7, Supporting Information).

**Figure 2:**
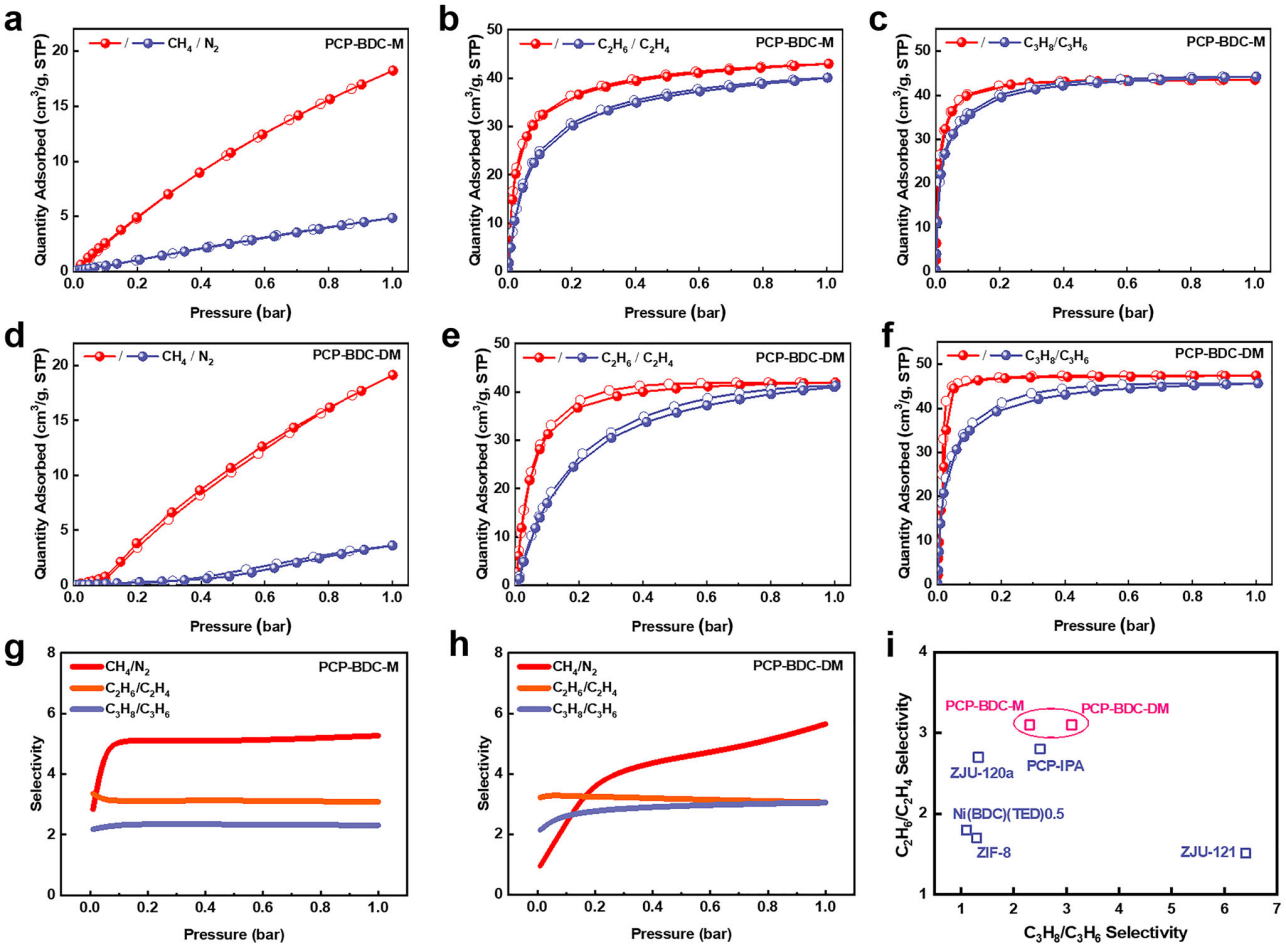
CH_4_/N_2_ a, d), C_2_H_6_/C_2_H_4_ b, e) and C_3_H_8_/C_3_H_6_ c, f) sorption isotherms at 298 K; IAST selectivity of CH_4_/N_2_, C_2_H_6_/C_2_H_4_ and C_3_H_8_/C_3_H_6_ g, h) on PCP‐BDC‐M and PCP‐BDC‐DM; C_2_H_6_/C_2_H_4_ selectivity plotted against C_3_H_8_/C_3_H_6_ selectivity at 298 K and 1 bar for some benchmark adsorbents i).^[^ ^29^, ^31^, ^32^, ^33^, ^34^ ^]^

To evaluate the adsorption performance of PCP‐BDC‐M and PCP‐BDC‐DM, adsorption isotherms for CH_4_/N_2_, C_2_H_6_/C_2_H_4,_ and C_3_H_8_/C_3_H_6_ were measured. Both PCP‐BDC‐M and PCP‐BDC‐DM exhibited alkane‐selective adsorption behavior. The CH_4_ and N_2_ adsorption isotherms on PCP‐BDC‐M and PCP‐BDC‐DM at 273 and 298 K are shown in **Figures** **2**a,d and S8 (Supporting Information). At pressures of 0.5 and 1 bar, PCP‐BDC‐M exhibited CH_4_/N_2_ uptake differences of 8.25 and 13.38 cm^3^ g^−1^, respectively, while PCP‐BDC‐DM showed slightly greater differences of 9.74 and 15.43 cm^3^ g^−1^ (Figures S9, Table S3, Supporting Information). Notably, at low pressure (0.1 bar), PCP‐BDC‐DM had a relatively low CH_4_ uptake (0.98 cm^3^ g^−1^), but adsorption significantly increased with rising pressure, reaching 19.15 cm^3^ g^−1^ at 1 bar, surpassing PCP‐BDC‐M. This phenomenon suggests PCP‐BDC‐DM can desorb CH_4_ at higher pressures, making it advantageous for efficient CH_4_ recovery in pressure swing adsorption (PSA) processes. Ideal Adsorbed Solution Theory (IAST) calculations indicated PCP‐BDC‐DM's CH_4_/N_2_ selectivity^[^ ^35^ ^]^ of 9.3 at 298 K and 1 bar, significantly exceeding PCP‐BDC‐M (5.3) and conventional adsorbents (Figure 2g,h; Table S4, Supporting Information).

Turning to the C_2_H_6_/C_2_H_4_ separation, both PCP‐BDC‐M and PCP‐BDC‐DM show the higher adsorption capacity for C_2_H_6_ compared to C_2_H_4_ (Figure 2b,e; Figures S10 and S12, Supporting Information), demonstrating a clear preferential adsorption of C_2_H_6_. At 298 K and 1 bar, both PCP‐BDC‐M and PCP‐BDC‐DM showed a C_2_H_6_/C_2_H_4_ selectivity of 3.1, surpassing most reported ethane‐selective adsorbents and also the parent PCP‐BDC material (2.8) (Figure S11, Supporting Information).^[^ ^36^ ^]^

The adsorption isotherms of C_3_H_8_ and C_3_H_6_ on PCP‐BDC‐M at 298 K showed that, in the low‐pressure region (< 0.3 bar), C_3_H_8_ exhibits slightly higher uptake than C_3_H_6_. However, as the pressure approaches 1 bar, the adsorption of C_3_H_8_ (43.50 cm^3^ g^−1^) becomes lower than that of C_3_H_6_ (44.23 cm^3^ g^−1^), indicating the limited capability for preferential adsorption of C_3_H_8_ over C_3_H_6_ (Figure 2c,f). In contrast, PCP‐BDC‐DM shows a more pronounced difference in adsorption between C_3_H_8_ and C_3_H_6_ across the entire pressure range, indicating its potential for superior C_3_H_8_/C_3_H_6_ separation performance. At 298 K and 1 bar, the C_3_H_8_/C_3_H_6_ selectivity of PCP‐BDC‐DM reaches 3.1, which is significantly higher than the 2.3 observed for PCP‐BDC‐M (Figure 2h and Figure S13, Supporting Information), and outperforms most reported propane‐selective adsorbents. The above results demonstrate that increased methyl substitution enhances PCP‐BDC‐DM's multifunctional alkane selectivity, significantly improving performance for CH_4_/N_2_, C_2_H_6_/C_2_H_4_, and C_3_H_8_/C_3_H_6_ separation (Table S5 and Figure 2i).

To further investigate the interactions between the adsorbates and the adsorbents, the isosteric heats of adsorption for various gases were calculated using the Virial equation^[^ ^37^ ^]^ as shown in Figures S14–S25 (Supporting Information). Both PCP‐BDC‐M and PCP‐BDC‐DM exhibited higher adsorption heats for light alkanes than their counterparts. Specifically, PCP‐BDC‐DM had notably higher adsorption enthalpies (CH_4_: 39.7 kJ mol^−1^; C_2_H_6_: 40.4 kJ mol^−1^; C_3_H_8_: 45.4 kJ mol^−1^) compared to PCP‐BDC‐M (CH_4_: 19.1 kJ mol^−1^; C_2_H_6_: 34.5 kJ mol^−1^; C_3_H_8_: 40.9 kJ mol^−1^), indicating that dimethyl functionalization significantly enhances these interactions.

**Figure 3:**
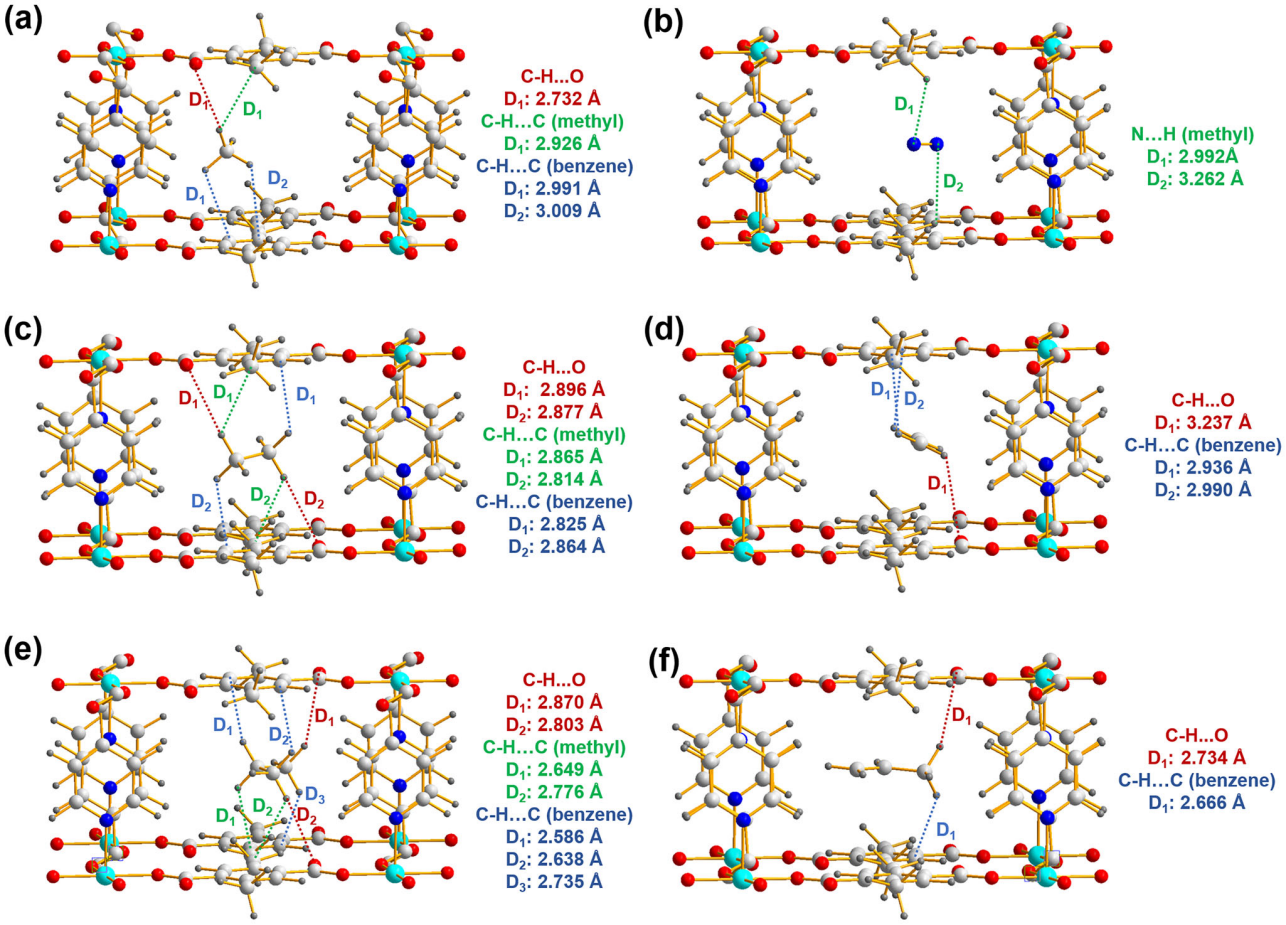
GCMC calculated preferable binding sites for paraffins and olefins in PCP‐BDC‐DM. CH_4_ a), N_2_ b), C_2_H_6_ c), C_2_H_4_ d), C_3_H_8_ e), and C_3_H_6_ f) binding sites in PCP‐BDC‐DM. The closest contacts between framework atoms and the gas molecules are defined by the distances (in Å), and the distances include the Van der Waals radius of the atoms. (Framework: C, white; H, gray; N, blue; O, red; Co, light blue; Gas: C, white; H, gray).

To gain molecular‐level insight into the adsorption behavior of CH_4_, N_2_, C_2_H_6_, C_2_H_4_, C_3_H_8_, and C_3_H_6_ within the channels of PCP‐BDC‐DM, Grand Canonical Monte Carlo (GCMC) simulations were performed, as illustrated in **Figure** **3**. The parallel alignment of terephthalic acid units, along with the presence of methyl groups, creates a tailored environment that favors interactions with the hydrogen atoms of alkanes. Specifically, the CH_4_ molecule engages in multiple van der Waals interactions with phenyl rings, the methyl group, and uncoordinated oxygen atoms: two C─H···C (phenyl ring) interactions with distances of 2.991–3.009 Å, one C─H···C (methyl group) interaction at 2.926 Å, and one C─H···O hydrogen bond at 2.732 Å) (Figure 3a). In contrast, the N_2_ molecule forms only two weak N···H (methyl) interactions (2.992–3.262 Å) with the framework (Figure 3b), indicating significantly weaker binding. Similarly, alkane exhibits denser and stronger interactions with the terephthalic acid units and methyl groups than olefins. Specifically, C_2_H_6_ and C_3_H_8_ molecules form multiple van der Waals interactions with the methyl groups (CH···C = 2.814–2.865 and 2.649–2.776 Å, respectively, Figure 3c,e). In contrast, C_2_H_4_ and C_3_H_6_ exhibit fewer and weaker interactions, mainly limited to C‐H···O hydrogen bond (3.237 and 2.734 Å) and C─H···C van der Waals contacts (2.936–2.990 and 2.666 Å), as shown in Figure 3d,f. These simulations affirm that methyl functionalities significantly enhance alkane binding, driving superior light alkane selectivity.

**Figure 4:**
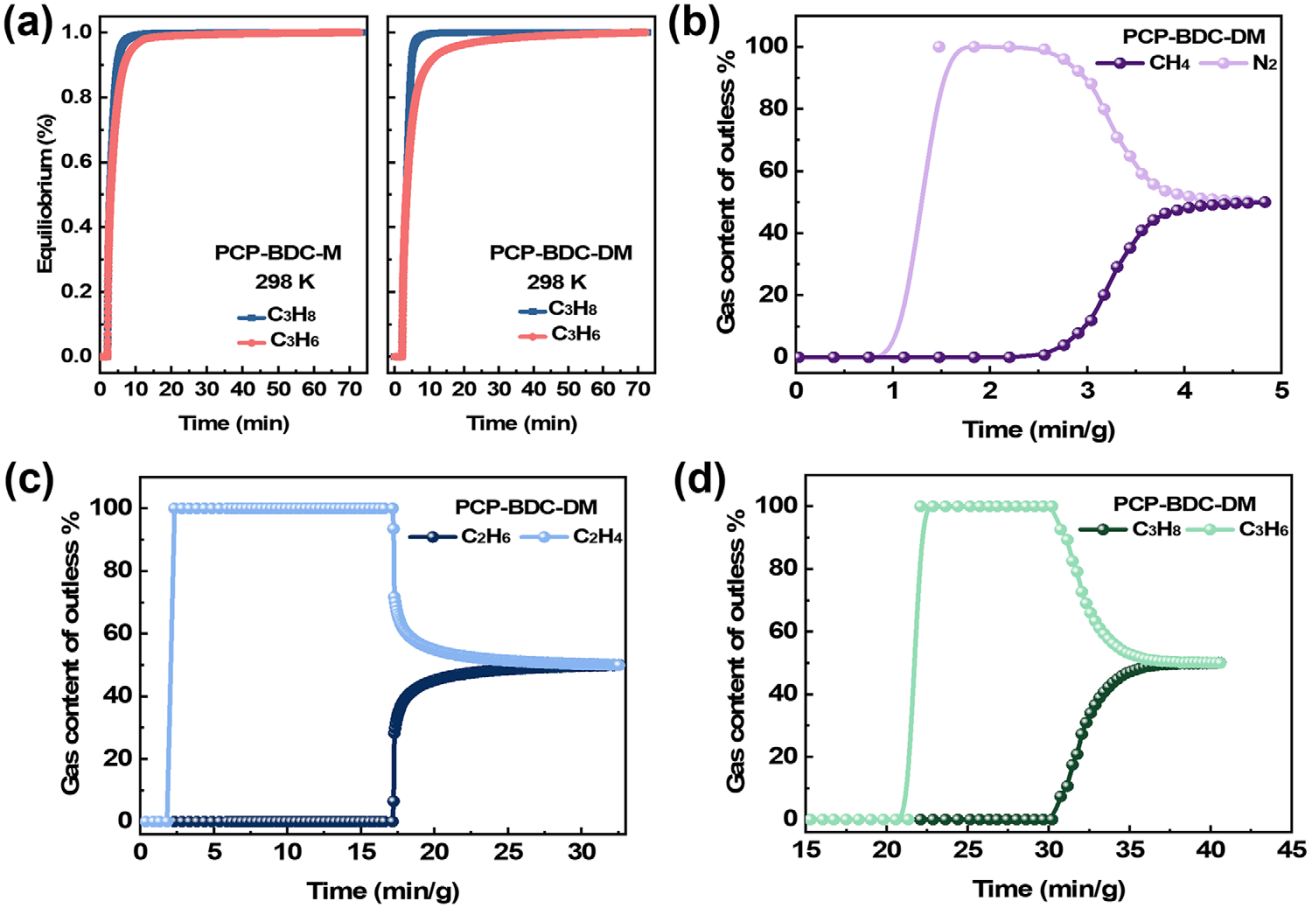
Adsorption kinetics of C_3_H_8_ and C_3_H_6_ on PCP‐BDC‐DM a), breakthrough curves of CH_4_/N_2_ (v/v = 50/50) at 15 mL min^−1^ b), C_2_H_6_/C_2_H_4_ (v/v = 50/50) at 15 mL min^−1^ and 298 K c), C_3_H_8_/C_3_H_6_ (v/v = 50/50) at 10 mL min^−1^ and 318 K on PCP‐BDC‐DM.

Adsorption kinetics for C_2_H_6_, C_2_H_4_, C_3_H_8_, and C_3_H_6_ were investigated (Figure 4a; Figure S26, Supporting Information). While C_2_H_6_ and C_2_H_4_ exhibited comparable kinetics, PCP‐BDC‐DM revealed substantial kinetic differences for C_3_H_8_ versus C_3_H_6_. C_3_H_8_ rapidly reached equilibrium (≈10 min), whereas C_3_H_6_ took longer (≈20 min). In competitive adsorption, faster kinetics of C_3_H_8_ enable it to dominate available adsorption sites, enhancing selectivity and separation efficiency. To further assess the separation performance under practical conditions, dynamic breakthrough experiments were conducted using equimolar (v/v = 50/50) gas mixture of CH_4_/N_2_, C_2_H_6_/C_2_H_4_, and C_3_H_8_/C_3_H_6_. Excellent separation efficiency was observed in all cases. For CH_4_/N_2_ separation on PCP‐BDC‐DM, N_2_ eluted first at ≈1.1 min g^−1^, while CH_4_ was retained until 2.6 min g^−1^ (Figure 4b), achieving a clear separation. In the case of the C_2_H_6_/C_2_H_4_ mixture separation, C_2_H_4_ consistently elutes earlier than C_2_H_6_ on both PCP‐BDC‐M and PCP‐BDC‐DM (Figure 4c and Figure S27, Supporting Information). Notably, PCP‐BDC‐DM exhibited superior separation performance, with a retention time of up to 15.3 min g^−1^ compared to 13.3 min g^−1^ on PCP‐BDC‐M. During this period, ultra‐high purity (>99.99%) C_2_H_4_ can be collected. Considering that the C_3_H_6_ product streams are typically released at elevated temperatures,^[^ ^38^ ^]^ breakthrough experiments for C_3_H_6_/C_3_H_8_ mixture were carried at 318 K with a flow rate of 10 mL min^−1^ to evaluate the real‐world separation performances of PCP‐BDC‐DM. In this system, C_3_H_6_ breaks through the column first, while C_3_H_8_ begins to elute only after 10 min g^−1^ (Figure 4d), confirming the material's excellent separation performance. The stability of the PCP‐BDC‐DM was studied. Adsorption isotherms at 298 K on PCP‐BDC‐DM of another batch and adsorption cycles test on PCP‐BDC‐DM were measured, showing the repeatability of adsorption (Figures S28 and S29, Supporting Information). In order to study the acid‐base and hydrothermal stability, PCP‐BDC‐DM was immersed in solutions of different pH values and in water at 80 °C for 12 h. The results show that within the pH range of 3 to 11 and in hot water, the structure of PCP‐BDC‐DM remains basically unchanged, indicating its excellent stability (Figure S30, Supporting Information). Then, PXRD of PCP‐BDC‐DM after breakthrough tests and cycle tests further confirmed structural integrity and stability (Figure S31, Supporting Information).

## 3 Conclusion

This study systematically examined the adsorption separation performance of light hydrocarbons on methyl‐functionalized PCP series adsorbents. Results indicate that methyl groups effectively create additional alkane adsorption sites, significantly enhancing separation selectivity across CH_4_/N_2_, C_2_H_6_/C_2_H_4_, and C_3_H_8_/C_3_H_6_ mixtures and creating a multifunctional adsorbent. Specifically, PCP‐BDC‐DM exhibited optimal performance due to its dimethyl groups. GCMC simulations corroborated experimental findings, clarifying that methyl groups serve as potent alkane‐binding sites. Breakthrough experiments validated PCP‐BDC‐DM's practical utility, highlighting its comprehensive alkane separation capabilities. This work provides valuable insights and strategies for designing advanced alkane‐selective adsorbents.

## Conflict of Interest

The authors declare no conflict of interest.

## Supporting information



Supporting Information

## Data Availability

The data that support the findings of this study are available in the supplementary material of this article.
