# Supplementary material for: Rational Design of a Multifunctional MOFs for Alkane‐Selective Gas Separation
Source: Adv Sci (Weinh). 2025 Oct 14;13(2):e16118. doi: 10.1002/advs.202516118 (PMC12786367; doi:10.1002/advs.202516118)
Supplement: Supplementary file 1 — Supporting Information [file ADVS-13-e16118-s001.docx]

**Supporting Information**

**Rational Design of a Multifunctional MOFs for Alkane-Selective Gas Separation**

Li Wang^a^, Zhaozhuang Liu^a^, Yating Wang^a^, Jiaqi Liu^b^^*^, Jinping Li^a, b^, Jiangfeng Yang^a*^

^a^ College of Chemistry and Chemical Engineering, Taiyuan University of Technology, Taiyuan 030024, Shanxi, P. R. China.

^b^ Shanxi Research Institute of Huairou Laboratory, Taiyuan 030032, P. R. China

**Experiment**

1. **Materials and Synthesis**

All chemicals and solvents were used without further purification.

PCP-BDC-M: Firstly, 162 mg dpg was dissolved in DMF/MeOH (1:1, 60 mL). Then, 136 mg 2-Methyl-1,4-benzenedicarboxylic acid and 218 mg Co(NO_3_)_2_·6H_2_O were added to the above solution. Then the mixture was heated at 80 °C for 24 hours to yield as-synthesized crystals of PCP-BDC-M and the sample was washed with DMF and MeOH three times respectively.

PCP-BDC-DM: Firstly, 162 mg dpg was dissolved in DMF/MeOH (1:1, 60 mL). Then, 136 mg 2,5-dimethylterephthalic acid and 218 mg Co(NO_3_)_2_·6H_2_O were added to the above solution. Then the mixture was heated at 80 °C for 24 hours to yield as-synthesized crystals of PCP-BDC-DM and the sample was washed with DMF and MeOH three times respectively.

1. **General Methods**

Powder X-ray diffraction (PXRD) patterns were recorded with Bruker D8 Advanced X-ray diffractometer with 2D array detector using Cu Kα radiation (λ = 1.54178 Å). The TGA curves were obtained from a NETZSCH analyzer. For each run, 3-5 mg sample was heated from room temperature to 800 ℃ at a ramp rate of 10 ℃/min.

**3. Single component gas adsorption measurements**

CO_2_, CH_4_, N_2_ adsorption isotherms were measured by ASAP 2460 (Micromeritics, America) gas adsorption instruments. C_2_H_6_, C_2_H_4_, C_3_H_8_ and C_3_H_6_ adsorption isotherms were measured by M660 (BSD instrument, China) gas adsorption instruments. Before all of gas sorption and breakthrough separation experiments, samples were activated at 180 °C under vacuum for overnight. The purity of gas for test was 99.999%

**4. Ideal adsorbed solution theory (IAST) selectivity**

IAST selectivity calculations of CH_4_/N_2_ (v/v=1/1), C_2_H_6_/C_2_H_4_ (v/v=1/1) and C_3_H_8_/C_3_H_6_ (v/v=1/1) mixture at different temperatures were performed based on the single gas adsorption isotherms. Firstly, in order to properly capture the isotherm inflections, the single gas adsorption isotherms of C_2_H_6_, C_2_H_4_, CH_4_ and N_2_ were fitted with the Dual-site Langmuir-Freundlich model.

$q=q_{A, sat} \frac{b_{A}p^{v_{A}}}{1+b_{A}p^{v_{A}}}+q_{B, sat} \frac{b_{B}p^{v_{B}}}{1+b_{B}p^{v_{B}}}$ (1)

In equation (1), q_A,sat_ and q_B,sat_ (mol/kg) are the saturated capacities of sites A and B, b*_A_* and b*_B_* are the Langmuir constants of sites A and B, and v*_A_* and v*_B_* are the Freundlich exponents of sites A and B, p (kPa) is the pressure of the bulk gas at equilibrium with the adsorbed phase (kPa), q (mol/kg) is the gas uptake amount of an adsorbent.

The single gas adsorption isotherms of C_3_H_8_ and C_3_H_6_ were fitted with the Dual-site Langmuir model.

$q=q_{A, sat} \frac{b_{A}p}{1+b_{A}p}+q_{B, sat} \frac{b_{B}p}{1+b_{B}p}$ (2)

In equation (2), the saturated capacities of sites A and B are represented by q_A,sat_ and q_B,sat_ (mol/kg) respectively. The Langmuir constants for sites A and B are denoted as b_A_ and b_B_. The pressure of the bulk gas at equilibrium with the adsorbed phase is indicated by p (kPa), while q (mol/kg) represents the amount of gas uptake by an adsorbent.

Then, the adsorption selectivity was calculated from

$S_{ads}=\frac{{q_{1}}/{q_{2}}}{{p_{1}}/{p_{2}}}$ (3)

In equation (3), q*_1_* and q*_2_* are the molar loadings in the adsorbed phase in equilibrium with the bulk gas phase with partial pressures p*_1_* and p*_2_*.

**5. Isosteric heat of adsorption**

To determine the affinity between the scaffold and the adsorbates, the Virial equation was employed to calculate the adsorption heat for CH_4_, N_2_, C_2_H_6_, C_2_H_4_, C_3_H_8_ and C_3_H_6_ which was defined as follows:

*Ln*(*P*)*=Ln*(*N*)*+*$\frac{\text{1}}{\text{T}}\sum_{\text{i=0}}^{\text{m}} \text{a}_{\text{i}}\text{N}_{\text{i}}$*+*$\sum_{\text{i=0}}^{\text{n}} \text{b}_{\text{i}}\text{N}_{\text{i}}$ *(4)*

*Q_st_=-R*$\sum_{i=0}^{m} a_{i}N_{i}$ *(5)*

where N is the adsorption amount, and m and n determine the number of items required to precisely fit the adsorption isotherms.

**6. PXRD refinement result**

The refined package is TOPAS V5.0, which describes the background using 12th-order Chebyshev polynomials and the peak shapes using pseudo-Voigt peak shapes. Cell parameters, zero displacement, atomic coordinates and occupancy, isotropic temperature factors were refined and the preferred orientation is described using the spherical cofunction of order 4. All parameters are liberally optimized to convergence.

**7.** **Grand canonical Monte Carlo (GCMC) simulations**

All simulations were performed by the Materials Studio (MS) 2020 package. The preferred sorption locations were performed by GCMC simulations with Adsorption fixed loading task and Metropolis method^1^ in the sorption calculation module. As for all of the GCMC simulations, the framework was considered to be rigid. The framework and gas molecule were described by the force filed of COMPASS Ⅲ. The cutoff radius was set to 12.5 Å, for the Lennard-Jones (LJ) interactions, and the electrostatic interactions, and the Ewald summation method was selected to calculate the electrostatic interactions between adsorbates as well as between adsorbates and the framework. For state point in GCMC simulation, the system adopted 1 × 10^6^ Monte Carlo steps to guarantee equilibration, and the ultimate data was collected for another 1 × 10^6^ Monte Carlo steps. The charges of the atoms of both gas molecules and the framework were assigned by the force filed of COMPASS Ⅲ.

**8. Adsorption kinetics**

Adsorption kinetics were measured with an Intelligent Gravimetric Analyzer (IGA001, Hiden, UK), which uses a gravimetric technique to accurately measure the gas sorption on materials under diverse operating conditions. The system was degassed until no further weight loss was observed. The system temperature was then reduced to room temperature and the sample pool was kept in a vacuum for 60 mins. For the gases considered, the adsorption equilibrium time of the sample at the pressure point (1 bar) was set for collection of the adsorption equilibrium data. Different gas adsorption kinetic tests were performed in accordance with the aforementioned steps. All the gases used (C_3_H_8_, C_3_H_6_, C_2_H_6_, C_2_H_4_ and He) were of 99.999% purity.

**9. Column Breakthrough Experiments**

The breakthrough experiments were carried out in a home-made dynamic mixed-gas breakthrough setup. In a typical experiment, 0.38 g of PCP-BDC-M or 0.60 g of PCP-BDC-DM sample were packed into a stainless-steel column with inner dimensions of ϕ = 4.5 mm. The mixed-gas flow and pressure were controlled by using pressure-control valves and mass flow controllers. Outlet effluent from the column was continuously monitored using mass spectrometry (MS, HPR-20, Hiden). The column packed with sample was first purged with a flow of He (20 mL·min-1) for 1 h at room temperature. The mixed-gas flow rate during the breakthrough process of CH_4_/N_2_ (v/v=1/1, 15 mL/min), C_2_H_6_/C_2_H_4_ (v/v=1/1, 15 mL/min) and C_3_H_8_/C_3_H_6_ (v/v=1/1, 10 mL/min). After the breakthrough experiment, the sample was regenerated under vacuum 10 hours for cycling measurement.

Figure S1. Le Bail fitting for PCP-BDC-M (Rp = 4.25%, Rwp = 5.67%, GoF = 1.25).

Figure S2. Le Bail fitting for PCP-BDC-DM (Rp = 2.64%, Rwp = 3.60%, GoF = 1.27).


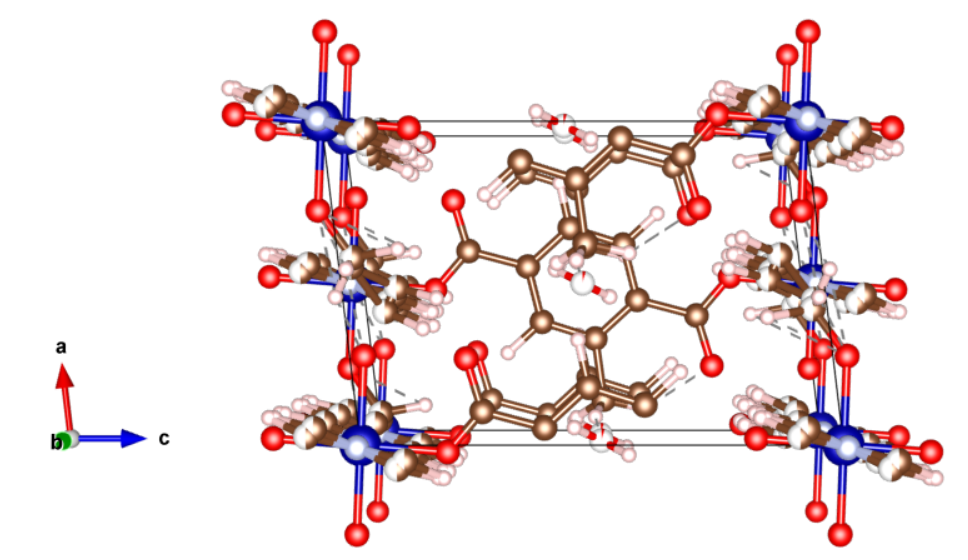


Figure S3. The structure picture of PCP-BDC-M.


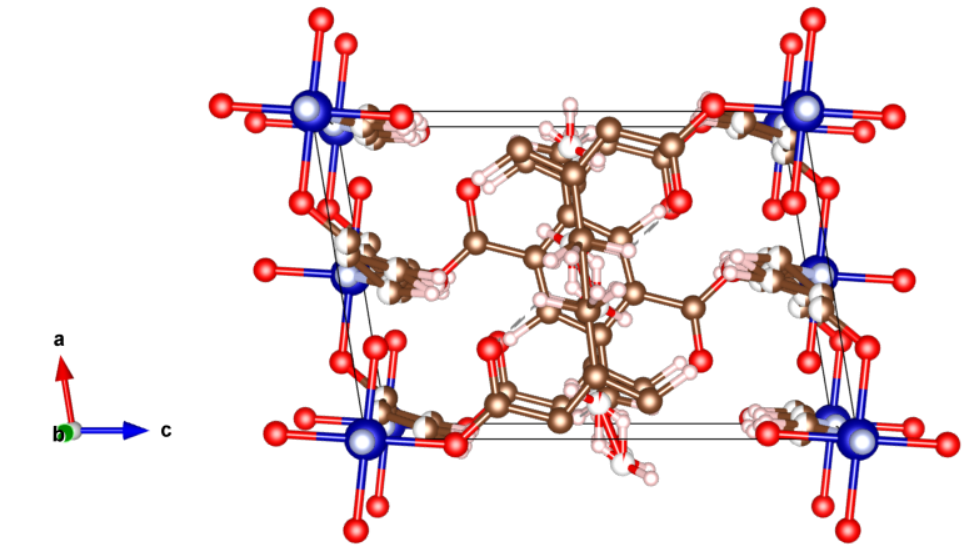


Figure S4. The structure picture of PCP-BDC-DM.

Table S1. Main parameters of processing refinement.

| compound | PCP-BDC-M | PCP-BDC-DM |
| --- | --- | --- |
| sp. gr. | C 2/m | C 2/m |
| a (Å) | 7.701 | 7.626 |
| b (Å) | 13.699 | 13.713 |
| c (Å) | 11.407 | 11.289 |
| β (°) | 96.865 | 99.434 |
| α, γ (°) | 90.0 | 90.0 |
| V (Å^3^) | 1194.824 | 1164.595 |
| 2θ-interval | 5°-40° | 5°-40° |
| no. of reflection | 66 | 63 |
| no. of refined parameters | 105 | 105 |
| R_wp_ (%) | 5.67 | 3.60 |
| R_p_ (%) | 4.25 | 2.64 |
| R_exp_ (%) | 4.54 | 2.83 |
| ꭓ^2^ | 1.25 | 1.27 |
| R_Br_ (%) | 1.02 | 0.96 |


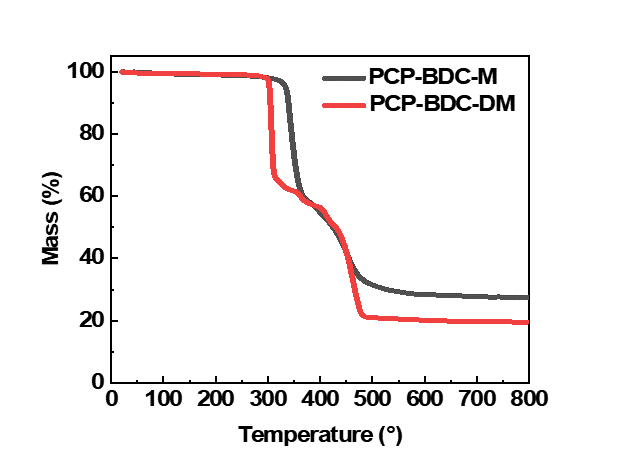


Figure S5. TGA curves of PCP-BDC-M and PCP-BDC-DM.


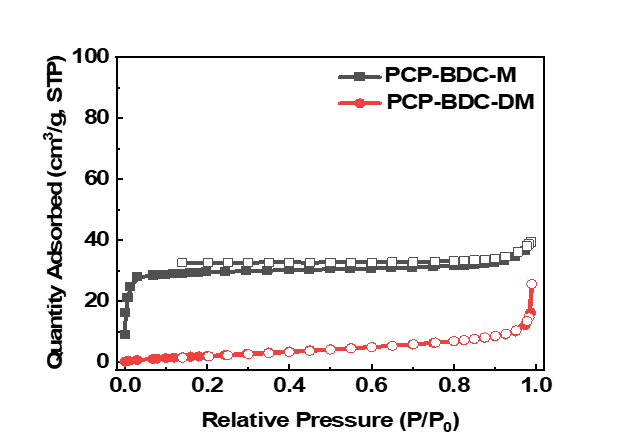


Figure S6. N_2_ sorption isotherms at 77 K on PCP-BDC-M and PCP-BDC-DM.


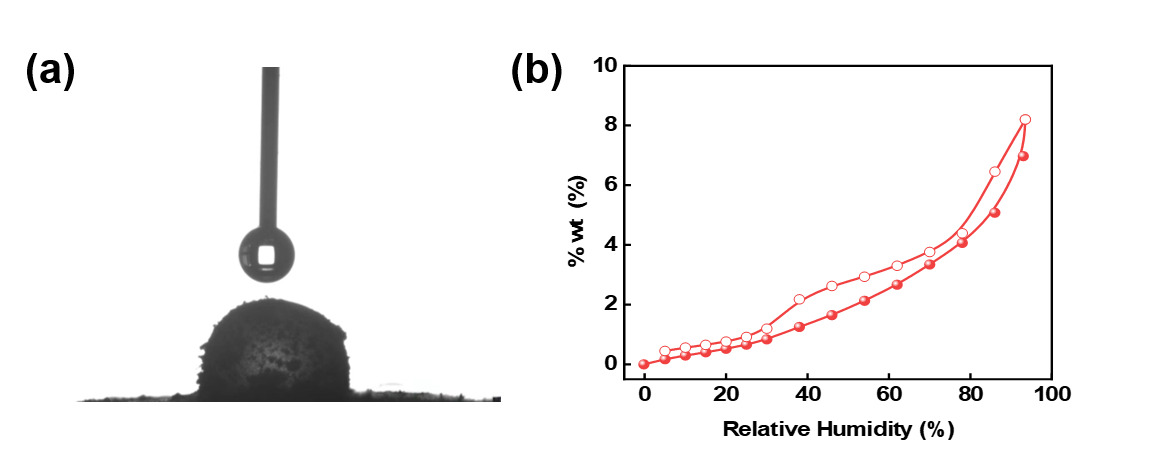


Figure S7. Contact angle test and water adsorption isotherms at 298 K on PCP-BDC-DM.

Table S2. The comparison of CO_2_ adsorption capacity, BET surface area and pore size on PCP-BDC-M and PCP-BDC-DM.

| Adsorbent | CO_2_ adsorption at 273 K (cm^3^/g) | Langmuir surface area (m^2^/g) | Pore size (Å) |
| --- | --- | --- | --- |
| PCP-BDC-M | 60.57 | 248.18 | 4.4 |
| PCP-BDC-DM | 47.67 | 186.49 | 4.2 |

Table S3. The comparison of CH_4_ adsorption at 298 K on PCP-BDC-M and PCP-BDC-DM.

| Adsorbent | CH_4_ uptake (cm^3^/g) | | | N_2_ uptake(cm^3^/g) | | |
| --- | --- | --- | --- | --- | --- | --- |
|  | 0.1 bar | 0.5 bar | 1 bar | 0.1 bar | 0.5 bar | 1 bar |
| PCP-BDC-M | 2.58 | 10.8 | 18.25 | 0.52 | 2.55 | 4.87 |
| PCP-BDC-DM | 0.98 | 10.81 | 19.15 | 0.2 | 1.07 | 3.72 |


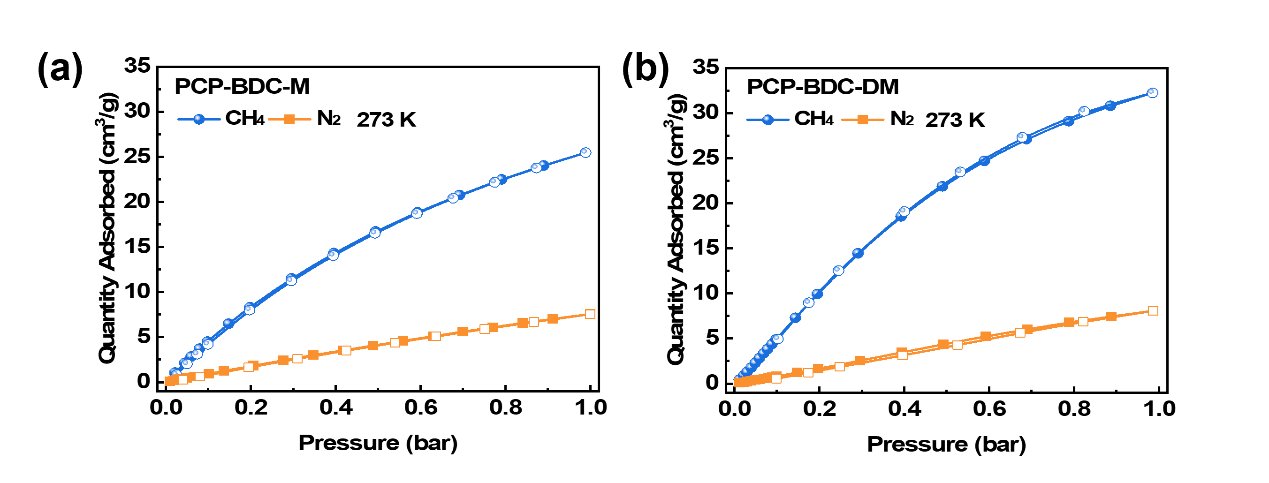


Figure S8. CH_4_ and N_2_ sorption isotherms at 273 K on PCP-BDC-M (a) and PCP-BDC-DM (b).


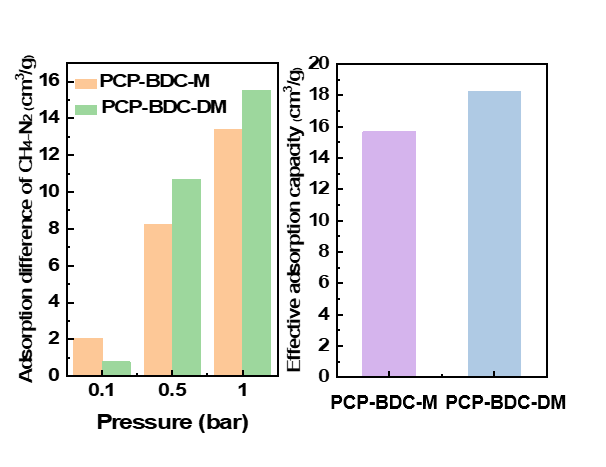


Figure S9. CH_4_ adsorption capacity at different pressures and comparison of efficient adsorption capacity on PCP-BDC-M and PCP-BDC-DM at 298 K.

Table S4. Summary of separation metrics of top-performing CH_4_/N_2_ separation materials reported in the literature at 1 bar and room temperature.

|  | CH_4_/N_2_ (50/50) selectivity | CH_4_ uptake (cm^3^/g)  298 K | N_2_ uptake (cm^3^/g)  298 K | Ref. |
| --- | --- | --- | --- | --- |
|  |  |  |  |  |
| Ni(ina)_2_ | 15.8 | 46.7 | 14.5 | 1 |
| Al-CDC | 13.1 | 32 | 5.1 | 2 |
| Co_3_(C_4_O_4_)_2_(OH)_2_ | 12.5 | 8.9 | 4.1 | 3 |
| CAU-21-BPDC | 11.9 | 22.2 | 4.2 | 4 |
| SBMOF-1 | 11.5 | 20.6 | 4 | 5 |
| ATC-Cu | 9.7 | 64.9 | 16.8 | 6 |
| STAM-1 | 10.8 | 14.2 | 2.4 | 7 |
| NKMOF-8-Me | 9 | 39.5 | 7 | 8 |
| MIL-160 | 8.9 | 10.5 | 3 | 9 |
| Al-FUM-Me | 8.6 | 27.2 | 5 | 9 |
| Cu(INA)_2_ | 8.3 | 18.6 | 2.7 | 10 |
| ZIF-94 | 7.4 | 33.6 | 8.2 | 11 |
| Ni-Qc-5-Dia | 7.4 | 29.3 | 6.2 | 12 |
| Ni-MA-BPY | 7.4 | 22.6 | 4.7 | 13 |
| Ni(BTC)(PIZ) | 7.3 | 36.3 | 7.8 | 14 |
| Co-MA-BPY | 7.2 | 20.6 | 4.4 | 13 |
| DMOF-A_2_ | 7.2 | 37 | 8.8 | 15 |
| CAU-10 | 7.2 | 16.6 | 5.3 | 9 |
| Ni(OAc)_2_L | 7 | 25.7 | 10.5 | 16 |
| Cu(hfipbb)(H_2_hfipbb)_0.5_ | 6.9 | 10.5 | 2.9 | 17 |
| TUT-100 | 6.3 | 27.5 | 5 | 18 |
| [Ni_3_(HCOO)_6_] | 6.2 | 18.4 | 4 | 19 |
| MIL-120Al | 6 | 33.7 | 10.5 | 20 |
| [Co_3_(HCOO)_6_] | 5.1 | 11 | 2.7 | 21 |
| MIL-53(Al) | 3.7 | 16.6 | 5 | 9 |
| ZIF-8 | 2.5 | 7.8 | 2 | 11 |
| PCP-BDC-M | 5.3 | 18.25 | 4.87 | This work |
| PCP-BDC-DM | 9.3 | 19.22 | 3.72 | This work |


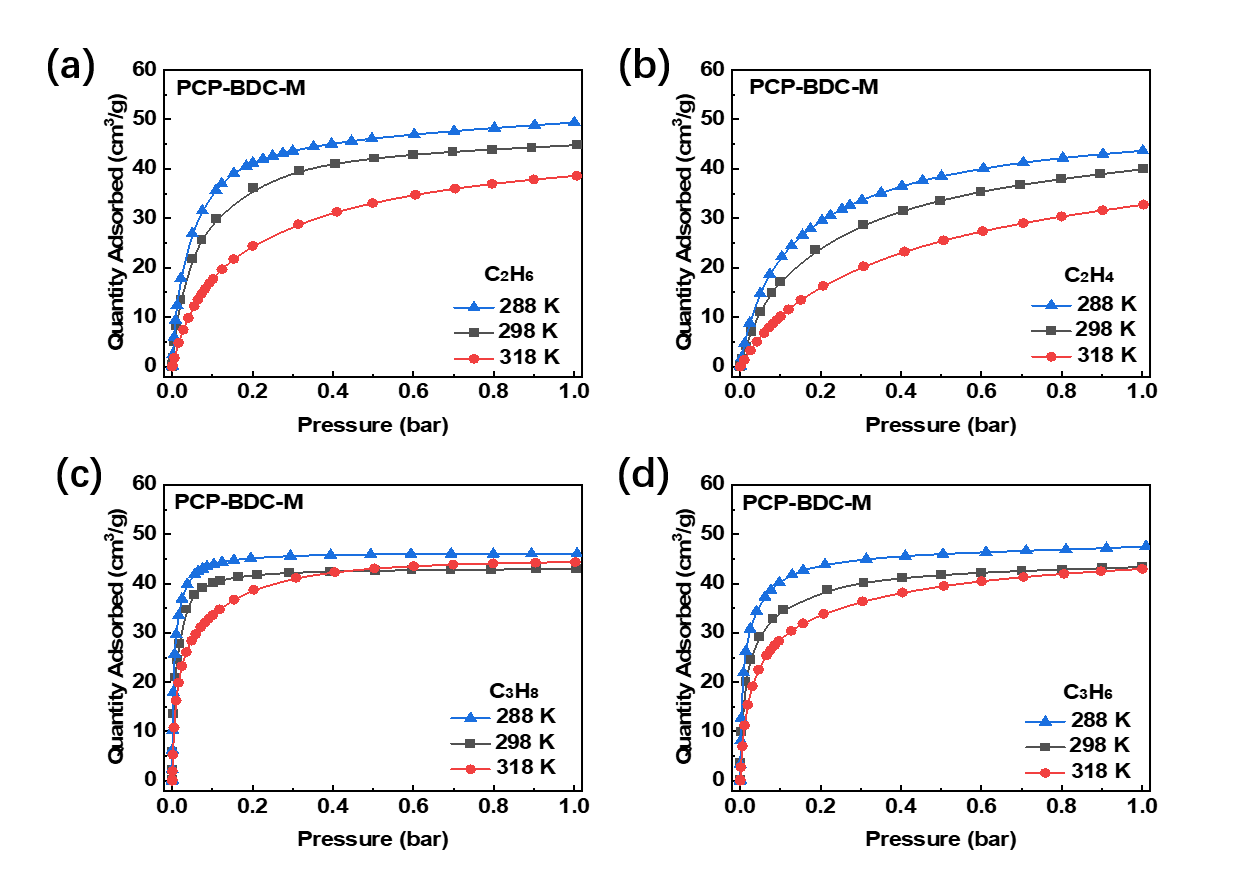


Figure S10. Adsorption isotherms of PCP-BDC-M at different temperature. C_2_H_6_ (a), C_2_H_4_ (b), C_3_H_8_ (c) and C_3_H_6_ (d).


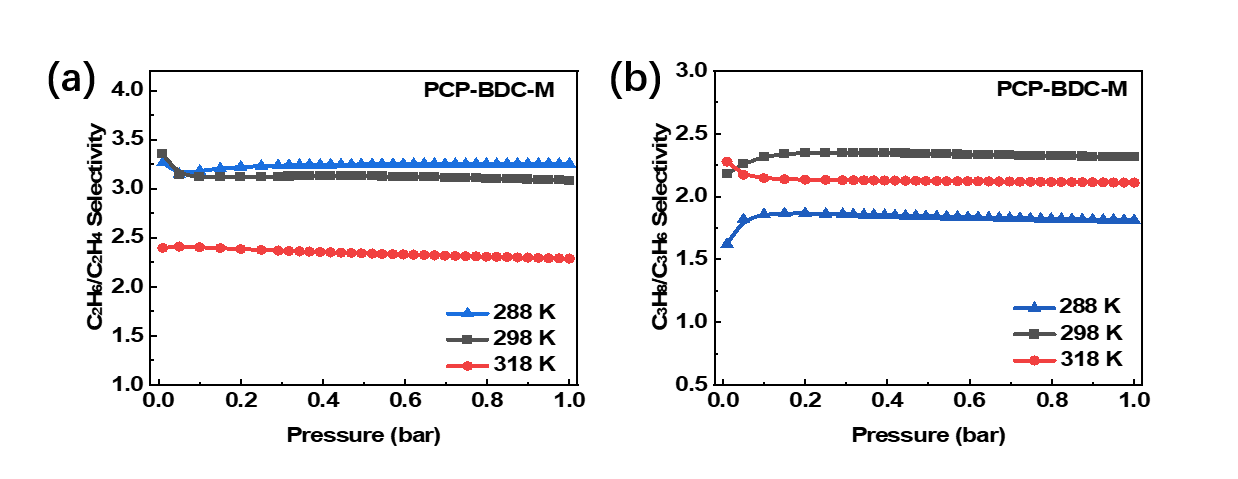


Figure S11. IAST selectivity of C_2_H_6_/C_2_H_4_ and C_3_H_8_/C_3_H_6_ on PCP-BDC-M at different temperatures.


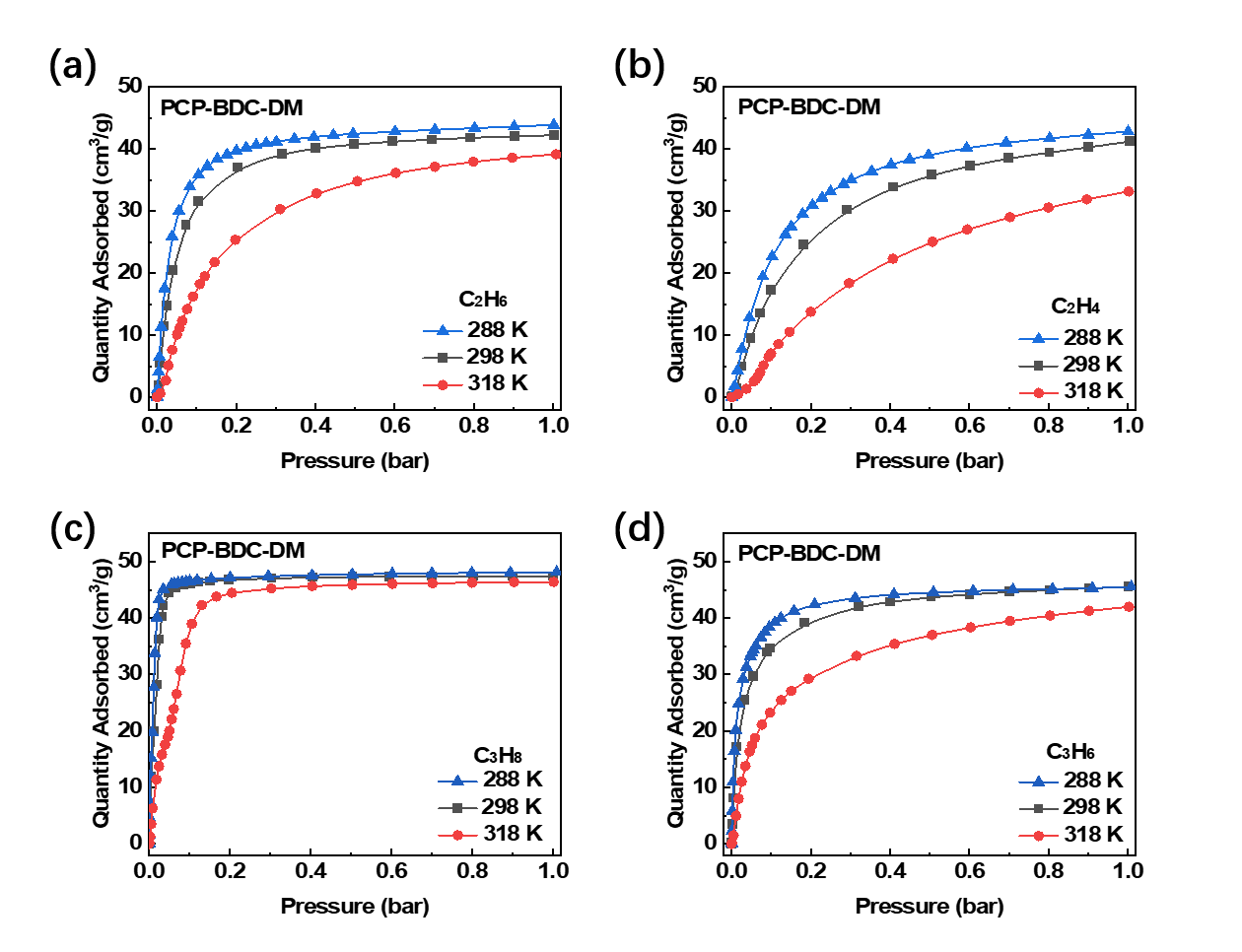


Figure S12. Adsorption isotherms of PCP-BDC-DM at different temperature. C_2_H_6_ (a), C_2_H_4_ (b), C_3_H_8_ (c) and C_3_H_6_ (d).


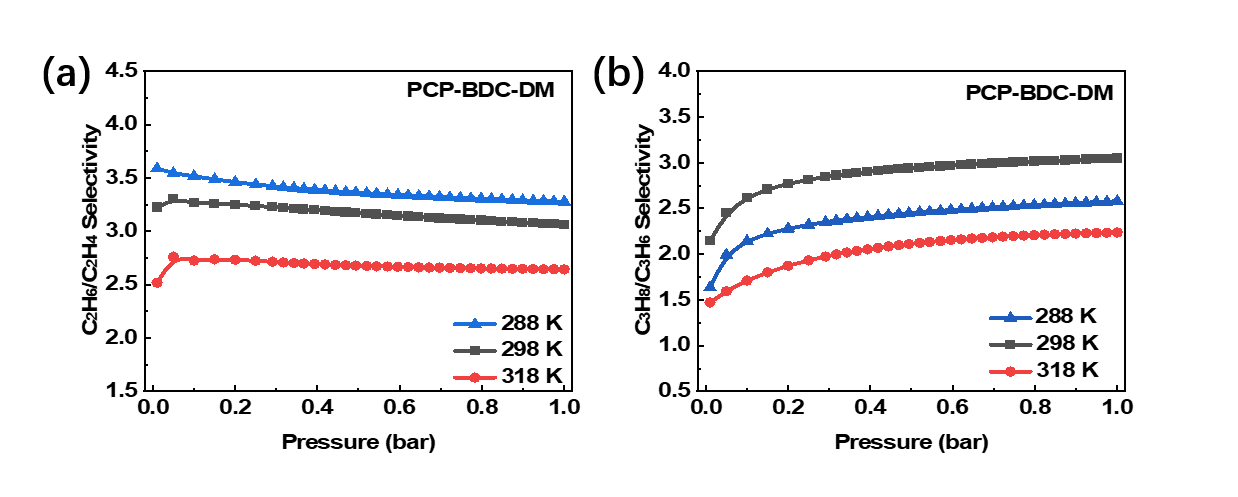


Figure S13. IAST selectivity of C_2_H_6_/C_2_H_4_ and C_3_H_8_/C_3_H_6_ on PCP-BDC-DM at different temperatures.

Table S5. Summary of separation metrics of top-performing ethane and propane-selective materials reported in the literature at 1 bar and room temperature

| Materials | C_2_H_6_ uptake (cm^3^/g) | C_2_H_4_ uptake (cm^3^/g) | C_3_H_8_ uptake (cm^3^/g) | C_3_H_6_ uptake (cm^3^/g) | C_2_H_6_/C_2_H_4_ selectivity | C_3_H_8_/C_3_H_6_ selectivity | Ref. |
| --- | --- | --- | --- | --- | --- | --- | --- |
| ZIF-8 | 56 | 32.03 | 87.81 | 91.84 | 1.7 | 1.3 | 22, 23 |
| ZJU-121 | 69.44 | 71.46 | 51.97 | 47.26 | 1.5 | 6.4 | 24, 25 |
| Ni(BDC)(TED)_0.5_ | 114.91 | 76.16 | 144.48 | 152.77 | 1.8 | 1.1 | 24, 25 |
| ZJU-120a | 110 | 88.03 | 120.96 | 123.87 | 2.7 | 1.3 | 24, 25 |
| PCP-IPA | 56 | 47.71 | 49.95 | 50.4 | 2.8 | 2.5 | 26 |
| PCP-BDC-M | 42.98 | 40.09 | 43.50 | 44.23 | 3.1 | 2.3 | This work |
| PCP-BDC-DM | 41.96 | 41.02 | 47.42 | 45.66 | 3.1 | 3.1 | This work |

Table S6. Dual-site Langmuir-Freundlich fitting parameters for C_2_H_6_, C_2_H_4_, CH_4_ and N_2_ in PCP-BDC-M and PCP-BDC-DM.

| Adsorbent | Gas | Temperature /K | q_A,sat_ | b_A_ |  | q_B,sat_ | b_B_ |  | R^2^ |
| --- | --- | --- | --- | --- | --- | --- | --- | --- | --- |
| PCP-BDC-M | C_2_H_6_ | 288 | 51.1521 | 17.5798 | 0.9129 | 1.2911 | 3.0889 | 8.0651 | 0.999 |
|  |  | 298 | 46.7265 | 13.2712 | 0.9147 | 1.2060 | 23500.6114 | 5.8609 | 0.999 |
|  |  | 318 | 16.6446 | 1.9555 | 1.1893 | 30.0861 | 10.9708 | 0.9995 | 0.999 |
|  | C_2_H_4_ | 288 | 31.2210 | 14.6099 | 0.9953 | 19.1159 | 3.0761 | 1.1770 | 0.999 |
|  |  | 298 | 23.5798 | 11.6816 | 1.0200 | 29.0329 | 1.6643 | 0.9280 | 0.999 |
|  |  | 318 | 30.2325 | 4.1877 | 0.9954 | 26.6803 | 0.4526 | 0.9963 | 0.999 |
| PCP-BDC-DM | C_2_H_6_ | 288 | 1.2911 | 3.0889 | 8.0651 | 51.1521 | 17.5798 | 0.9129 | 0.999 |
|  |  | 298 | 46.7265 | 13.2712 | 0.9147 | 1.2060 | 23500.6096 | 5.8609 | 0.999 |
|  |  | 318 | 30.0861 | 10.9708 | 0.9995 | 16.6446 | 1.9555 | 1.1893 | 0.999 |
|  | C_2_H_4_ | 288 | 31.2210 | 14.6099 | 0.9953 | 19.1159 | 3.0761 | 1.1770 | 0.999 |
|  |  | 298 | 23.5798 | 11.6816 | 1.0200 | 29.0329 | 1.6643 | 0.9280 | 0.999 |
|  |  | 318 | 26.6804 | 0.4526 | 0.9963 | 30.2324 | 4.1877 | 0.9954 | 0.999 |
| PCP-BDC-M | CH_4_ | 298 | 0.2004 | 75.9436 | 3.7440 | 63.5359 | 0.3960 | 0.9644 | 0.999 |
|  | N_2_ |  | 25.8523 | 0.2249 | 1.0972 | 19.1248 | 0.0066 | 0.2875 | 0.999 |
| PCP-BDC-DM | CH_4_ |  | 2.3895 | 9507.2051 | 5.2945 | 31.0147 | 1.1610 | 1.6460 | 0.999 |
|  | N_2_ |  | 0.5718 | 5.3866 | 1.1782 | 4.1345 | 3.0761 | 4.5390 | 0.999 |

Table S7. Dual-site Langmuir fitting parameters for C_3_H_8_ and C_3_H_6_ in PCP-BDC-M and PCP-BDC-DM.

| Adsorbent | Gas | Temperature /K | q_A,sat_ | b_A_ | q_B,sat_ | b_B_ | R^2^ |
| --- | --- | --- | --- | --- | --- | --- | --- |
| PCP-BDC-M | C_3_H_8_ | 288 | 28.5380 | 0.0026 | 18.0626 | 0.0141 | 0.999 |
|  |  | 298 | 9.5295 | 0.0012 | 34.1343 | 0.0130 | 0.999 |
|  |  | 318 | 23.7476 | 0.0066 | 23.2881 | 0.1023 | 0.999 |
|  | C_3_H_6_ | 288 | 26.8152 | 0.0033 | 21.4647 | 0.0500 | 0.999 |
|  |  | 298 | 25.8668 | 0.0068 | 19.2017 | 0.0917 | 0.999 |
|  |  | 318 | 20.4112 | 0.3033 | 27.7531 | 0.0179 | 0.999 |
| PCP-BDC-DM | C_3_H_8_ | 288 | 46.7280 | 0.0082 | 3.2613 | 0.0082 | 0.999 |
|  |  | 298 | 0.0744 | 0.0119 | 49.7208 | 0.0119 | 0.999 |
|  |  | 318 | 0.0147 | 0.0603 | 52.1680 | 0.0603 | 0.999 |
|  | C_3_H_6_ | 288 | 26.0394 | 0.0064 | 20.5548 | 0.0456 | 0.999 |
|  |  | 298 | 11.9812 | 0.1720 | 35.9345 | 0.0177 | 0.999 |
|  |  | 318 | 31.2300 | 2.9525 | 36.2927 | 0.0612 | 0.999 |


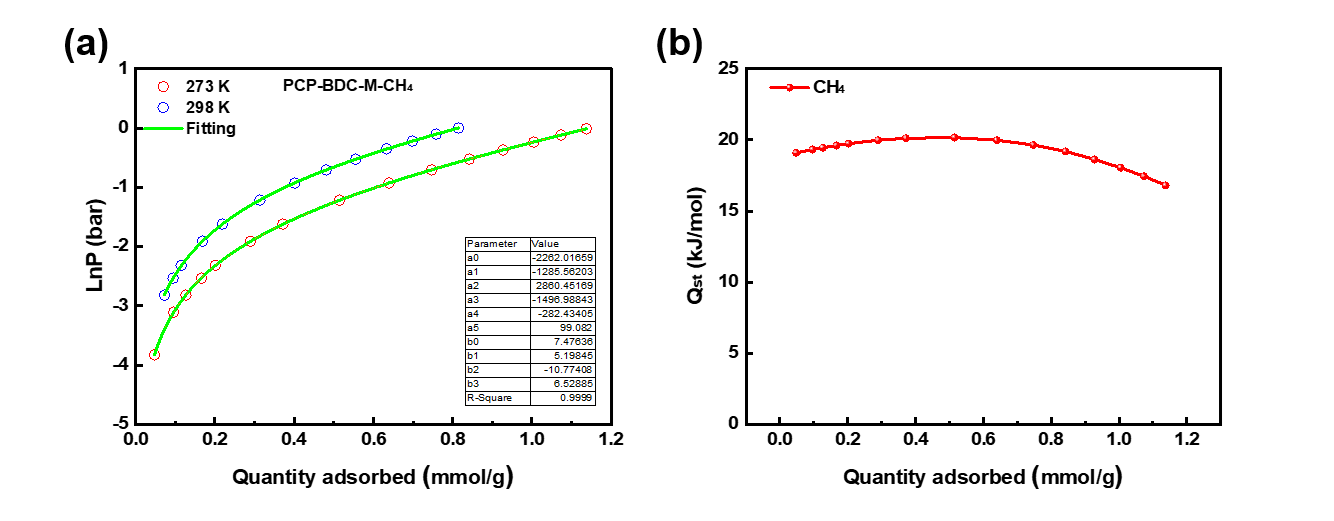


Figure S14. Fitting curves of CH_4_ adsorption isotherms on PCP-BDC-M at different temperature (a) and adsorption heats of CH_4_.


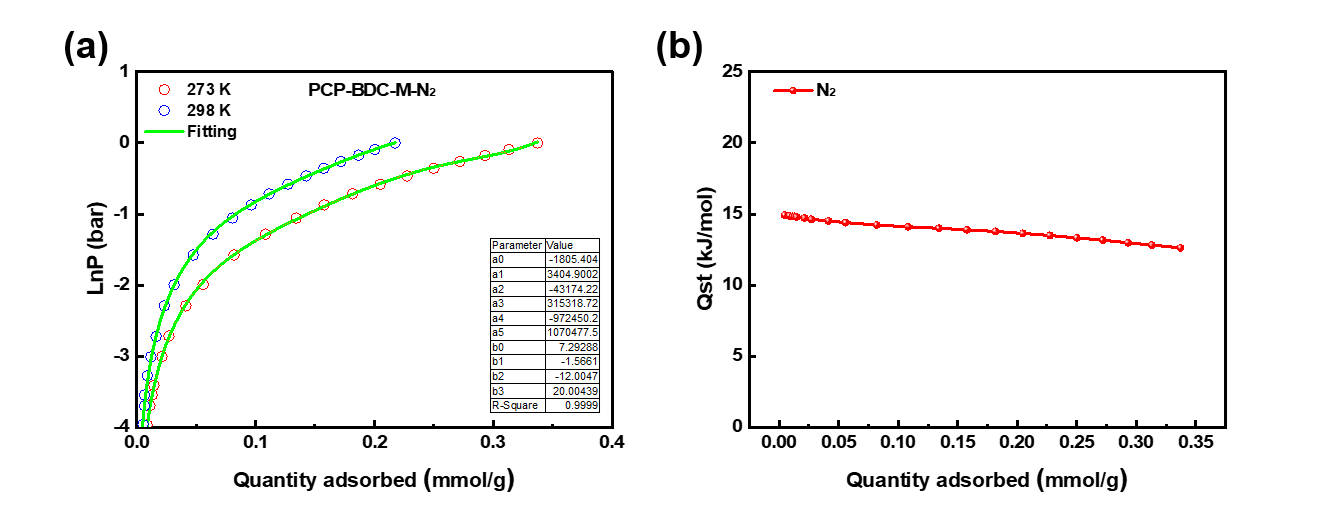


Figure S15. Fitting curves of N_2_ adsorption isotherms on PCP-BDC-M at different temperature (a) and adsorption heats of N_2_.


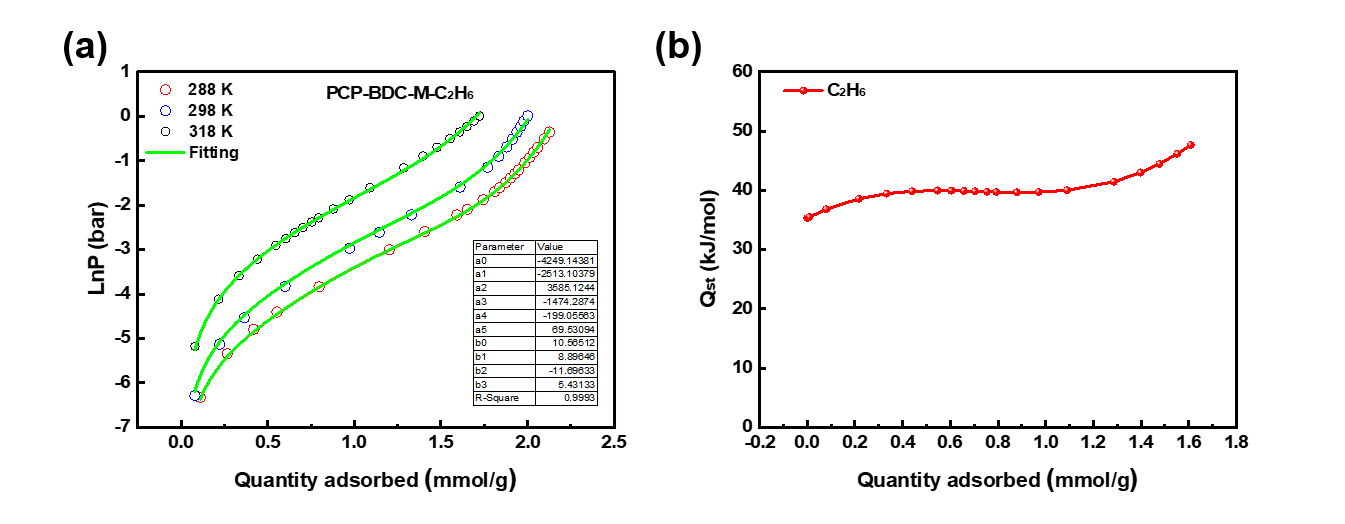


Figure S16. Fitting curves of C_2_H_6_ adsorption isotherms on PCP-BDC-M at different temperature (a) and adsorption heats of C_2_H_6_.


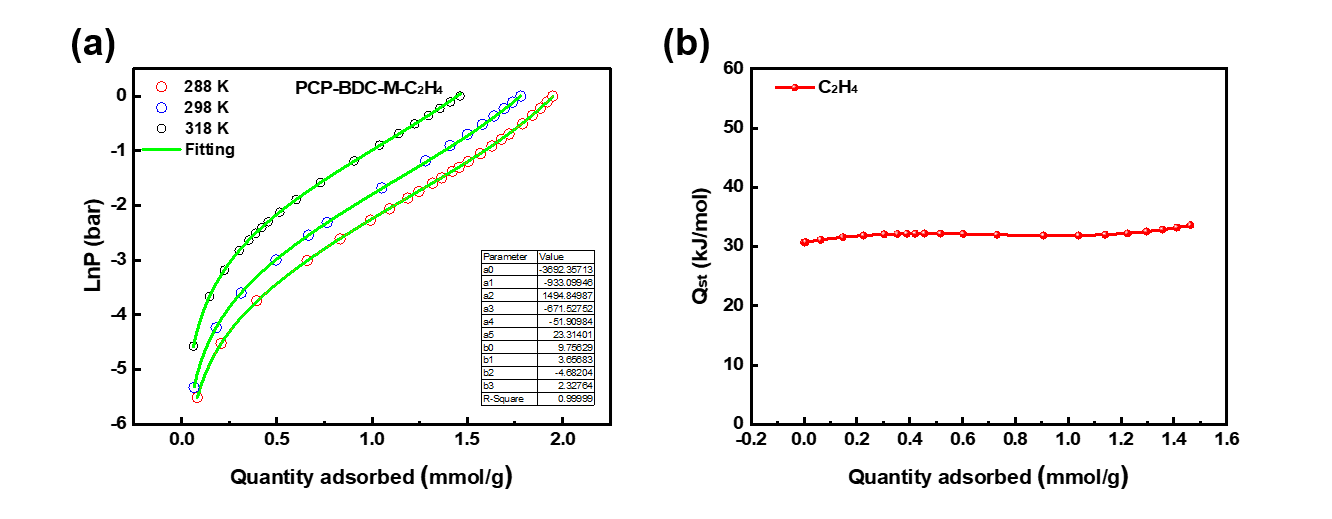


Figure S17. Fitting curves of C_2_H_4_ adsorption isotherms on PCP-BDC-M at different temperature (a) and adsorption heats of C_2_H_4_.


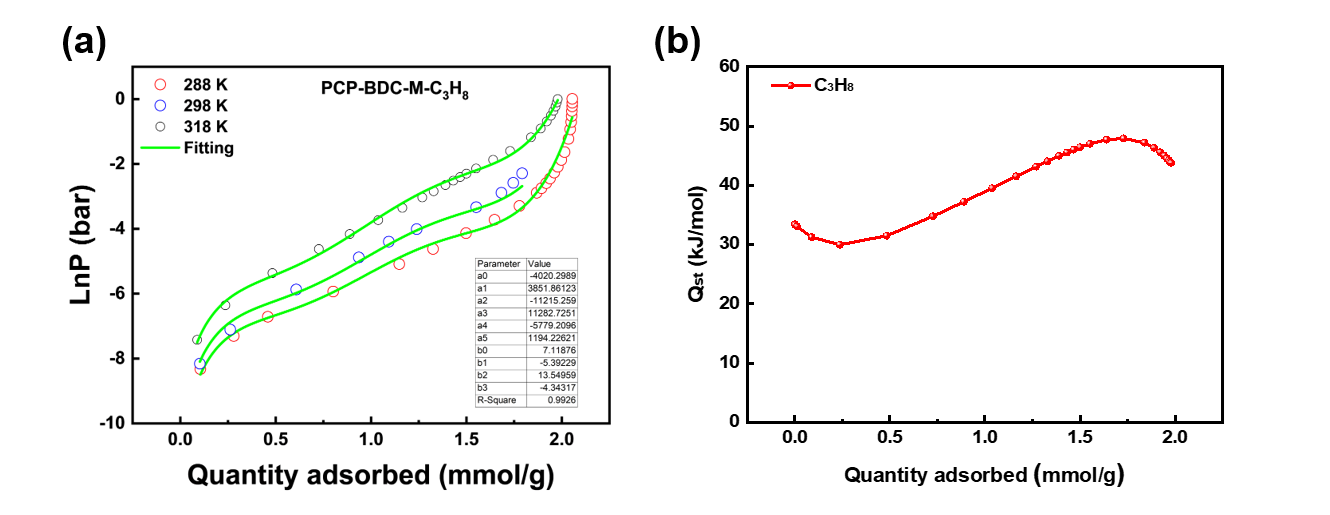


Figure S18. Fitting curves of C_3_H_8_ adsorption isotherms on PCP-BDC-M at different temperature (a) and adsorption heats of C_3_H_8_.


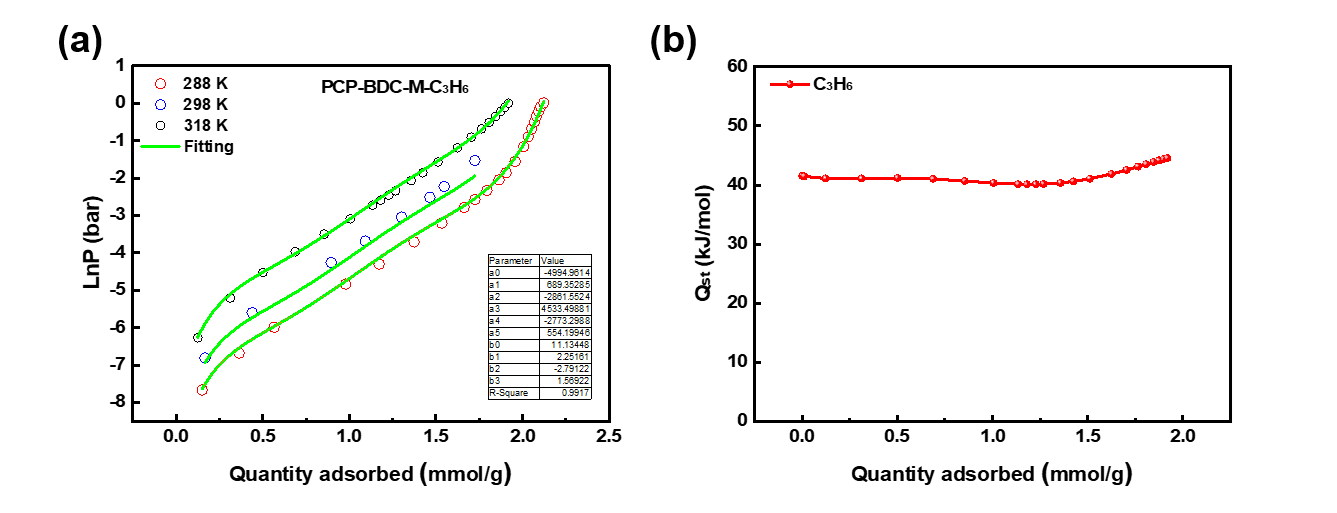


Figure S19. Fitting curves of C_3_H_6_ adsorption isotherms on PCP-BDC-M at different temperature (a) and adsorption heats of C_3_H_6_.


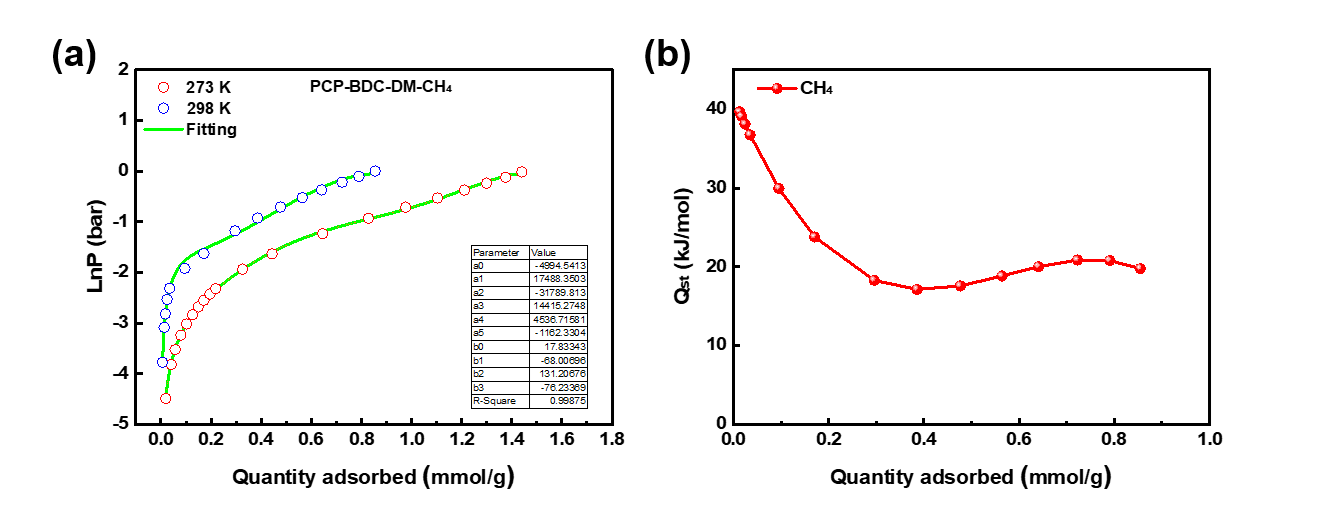


Figure S20. Fitting curves of CH_4_ adsorption isotherms on PCP-BDC-DM at different temperature (a) and adsorption heats of CH_4_.


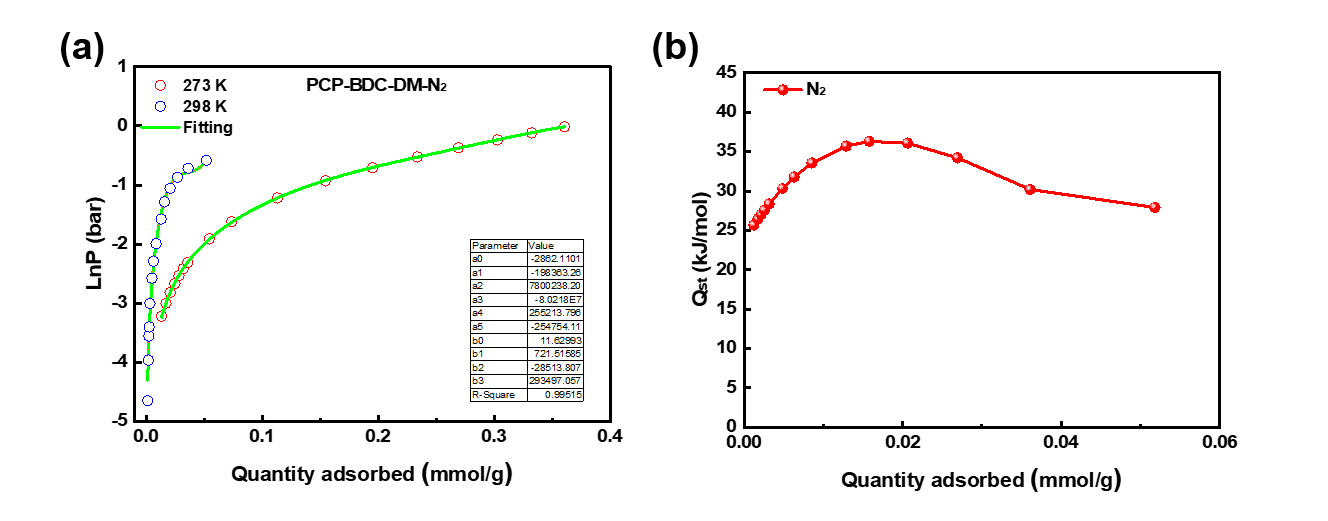


Figure S21. Fitting curves of N_2_ adsorption isotherms on PCP-BDC-DM at different temperature (a) and adsorption heats of N_2_.


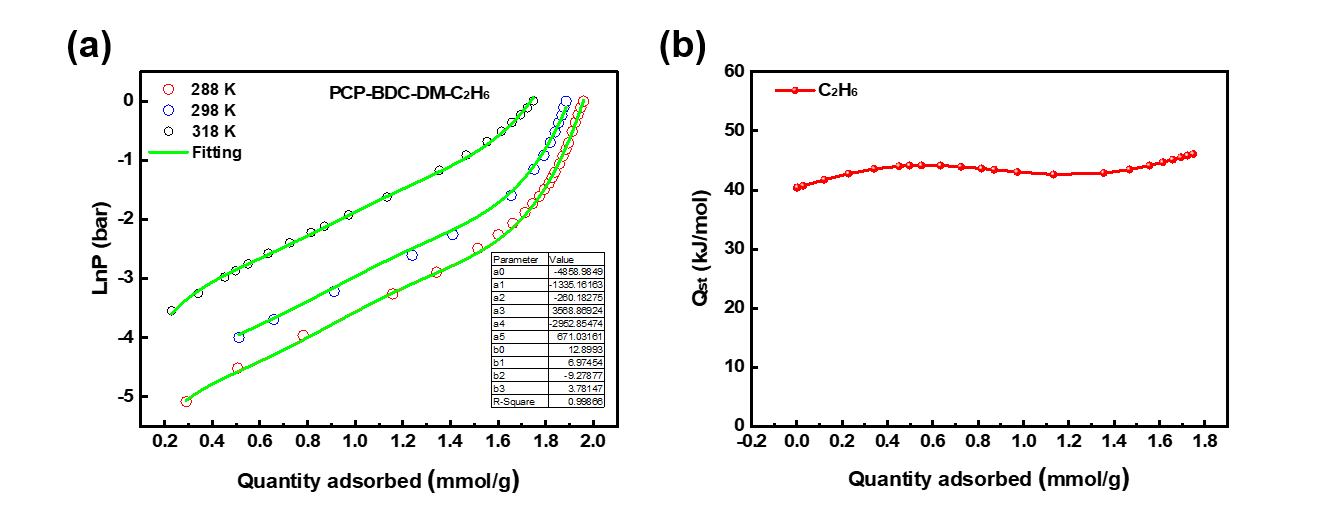


Figure S22. Fitting curves of C_2_H_6_ adsorption isotherms on PCP-BDC-DM at different temperature (a) and adsorption heats of C_2_H_6_.


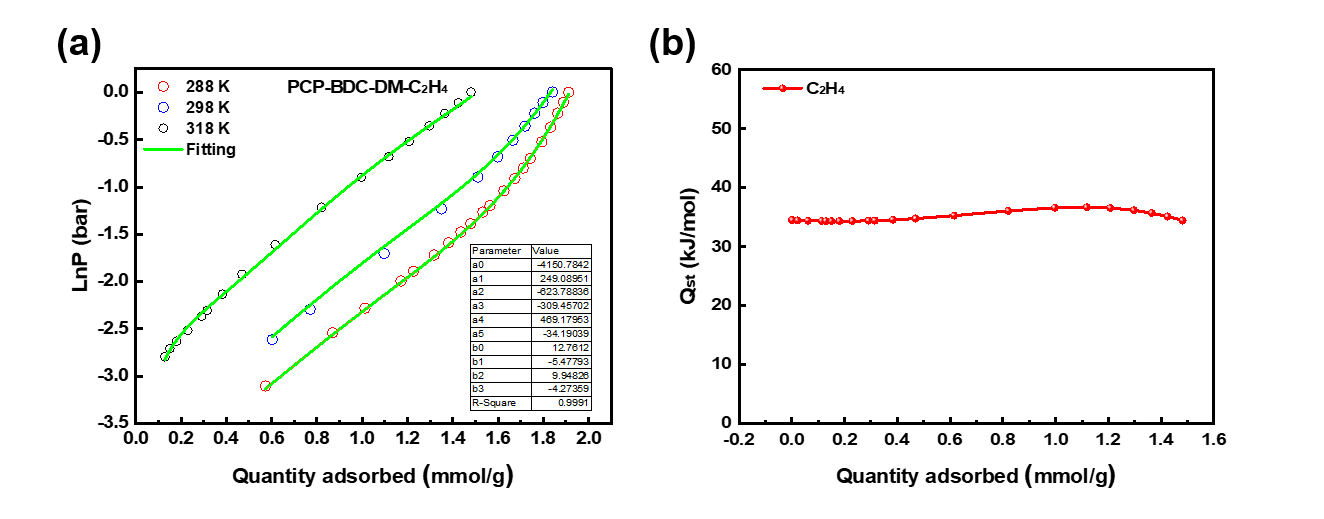


Figure S23. Fitting curves of C_2_H_4_ adsorption isotherms on PCP-BDC-DM at different temperature (a) and adsorption heats of C_2_H_4_.


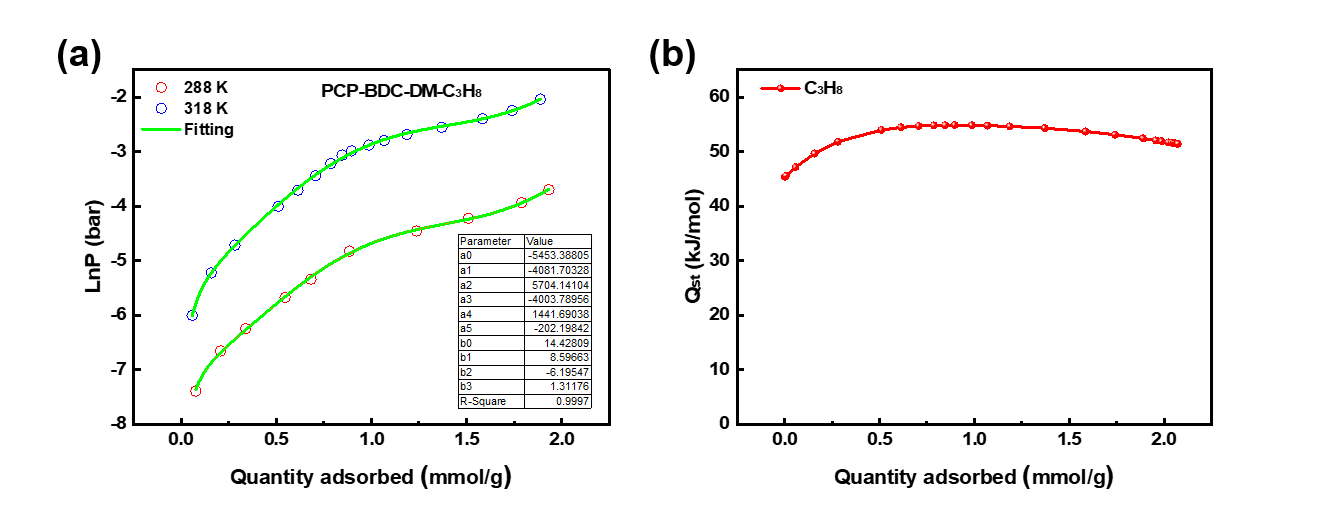


Figure S24. Fitting curves of C_3_H_8_ adsorption isotherms on PCP-BDC-DM at different temperature (a) and adsorption heats of C_3_H_8_.


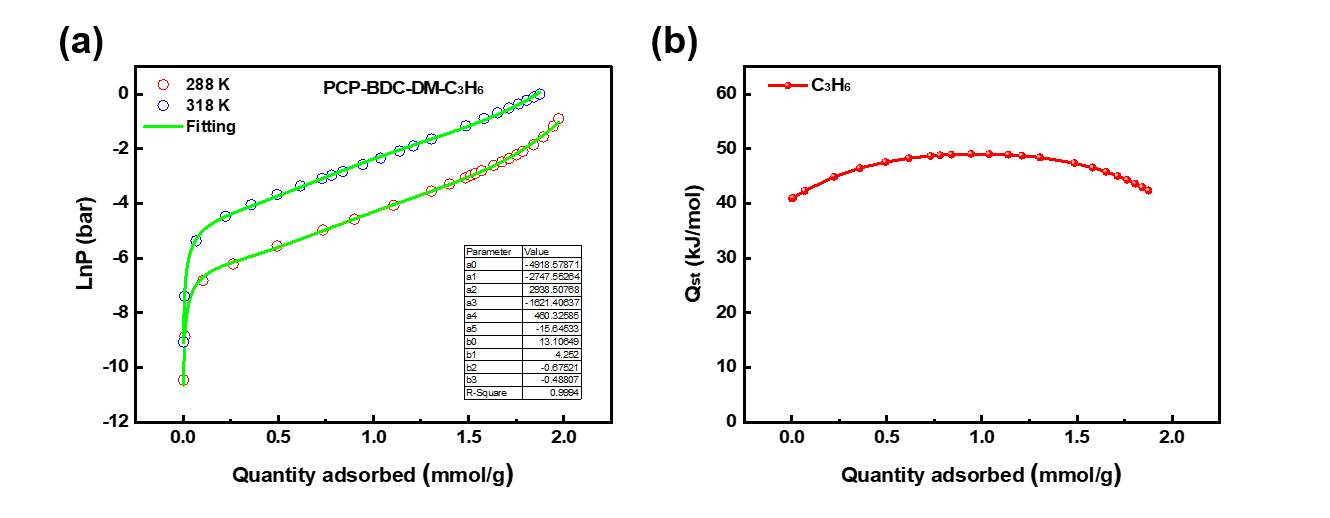


Figure S25. Fitting curves of C_3_H_6_ adsorption isotherms on PCP-BDC-DM at different temperature (a) and adsorption heats of C_3_H_6_.


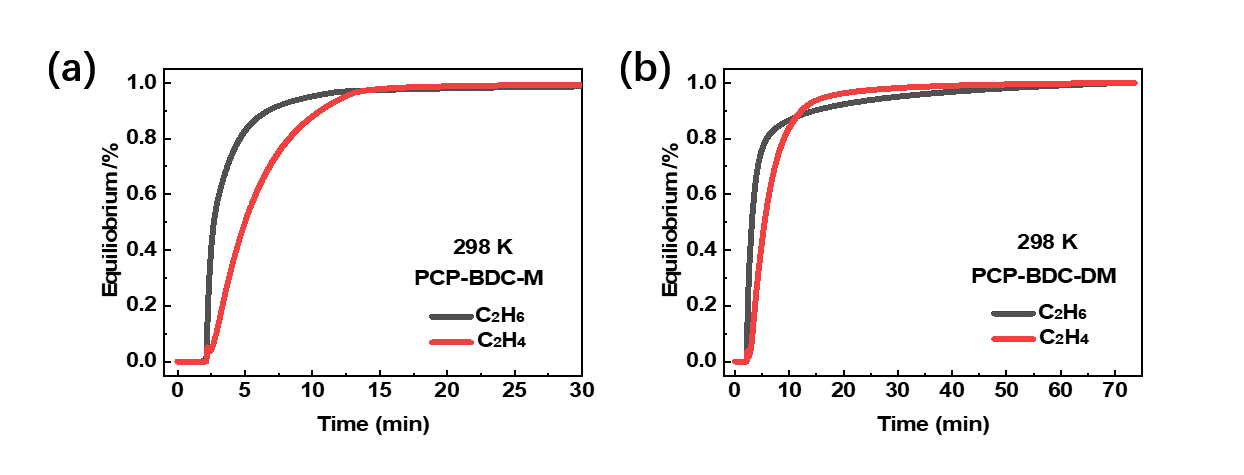


Figure S26. Adsorption kinetics of C_2_H_6_ and C_2_H_4_ at 298 K on PCP-BDC-M and PCP-BDC-DM.


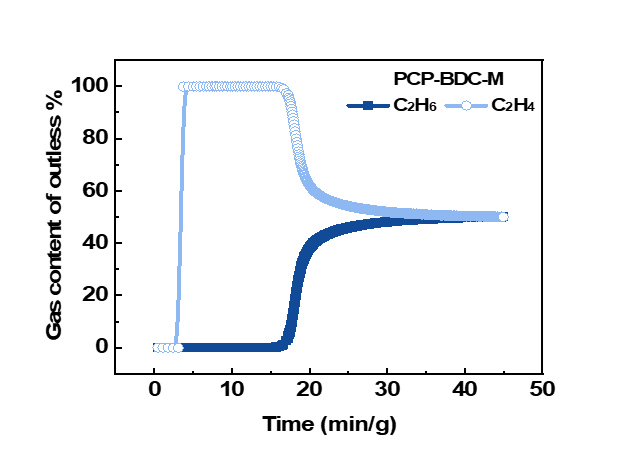


Figure S27. Breakthrough curve of C_2_H_6_ and C_2_H_4_ at 298 K and 15 mL/min on PCP-BDC-M.


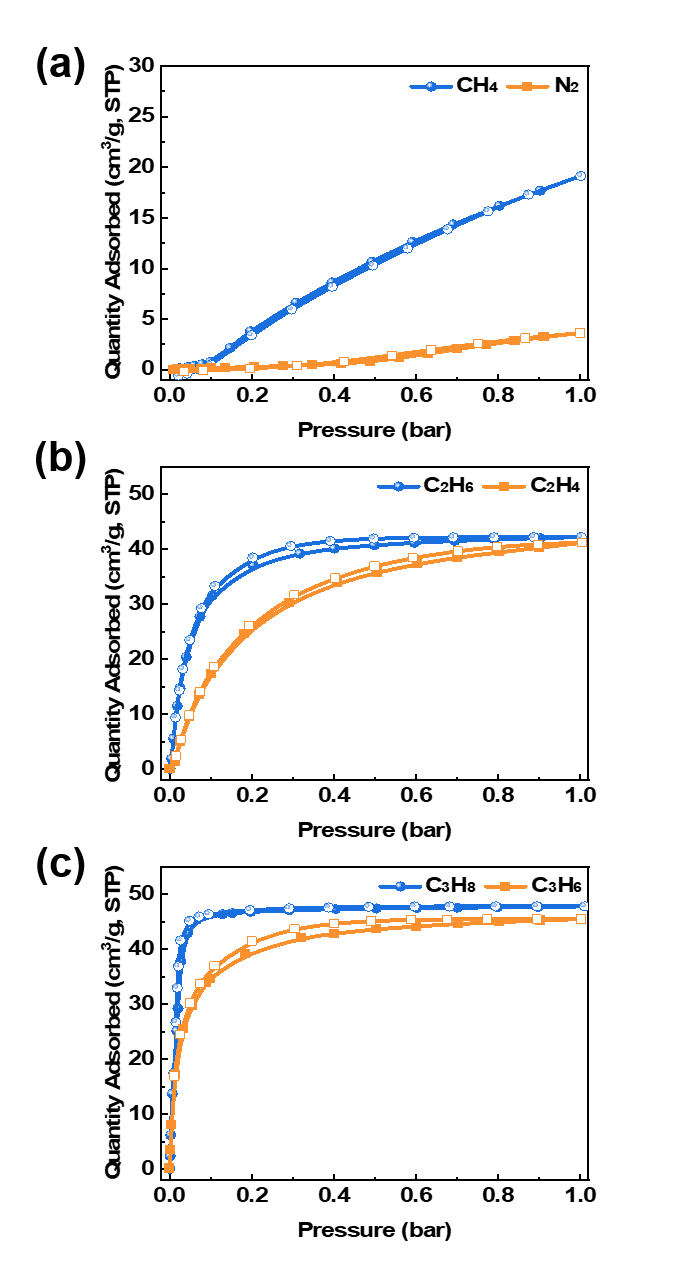


Figure S28. CH_4_, N_2_, C_2_H_6_, C_2_H_4_, C_3_H_8_ and C_3_H_6_ adsorption isotherms at 298 K on PCP-BDC-DM of another batch of samples.


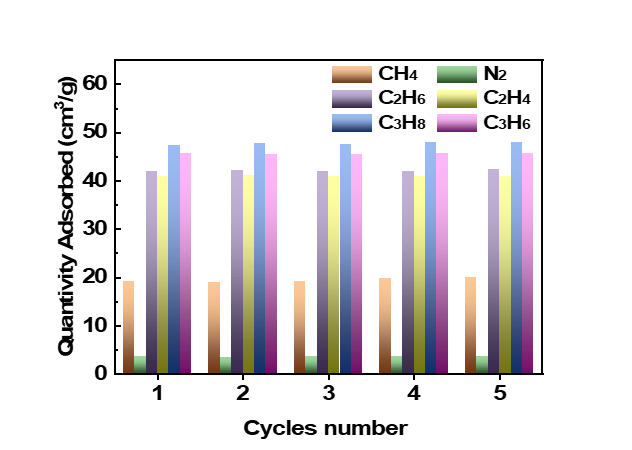


Figure S29. Adsorption cycles test on PCP-BDC-DM.


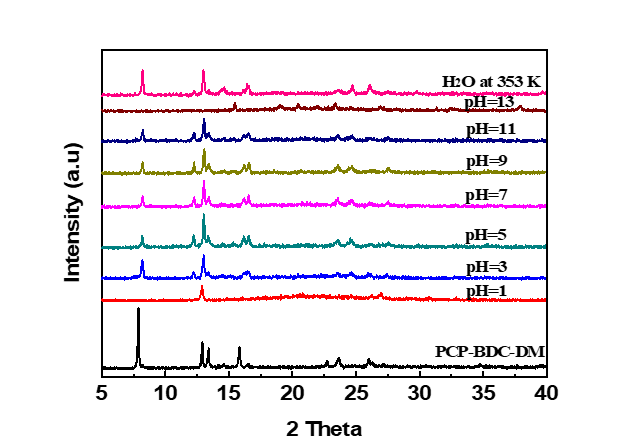


Figure S30. PXRD of PCP-BDC-DM soaked in different pH values and in water at 353 K for 12 h.


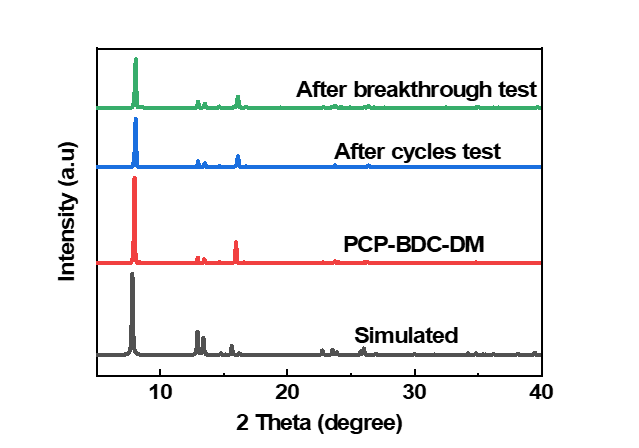


Figure S31. PXRD of PCP-BDC-DM after adsorption cycles and breakthrough test.

**Reference**

1. L. Li, L. Yang, J. Wang, Z. Zhang, Q. Yang, Y. Yang, Q. Ren, Z. Bao, *AIChE Journal* **2018**, 64, 3681.

2. D. Lv, Y. Wu, J. Chen, Y. Tu, Y. Yuan, H. Wu, Y. Chen, B. Liu, H. Xi, Z. Li, *AIChE Journal* **2020**, 66, e16287.

3. M. Chang, J. Ren, Q. Yang, D. Liu, *Chemical Engineering Journal* **2021**, 408, 127294.

4. Z. Niu, X. Cui, T. Pham, P. C. Lan, H. Xing, K. A. Forrest, L. Wojtas, B. Space, S. Ma, *Angewandte Chemie International Edition* **2019**, 58, 10138.

5. M. Chang, Y. Zhao, Q. Yang, D. Liu, *Acs Omega* **2019**, 4, 14511.

6. M. Chang, F. Wang, Y. Wei, Q. Yang, J. X. Wang, D. Liu, J. F. Chen, *AIChE Journal* **2022**, 68, e17794.

7. Z. Huang, P. Hu, J. Liu, F. Shen, Y. Zhang, K. Chai, Y. Ying, C. Kang, Z. Zhang, H. Ji, *Separation and Purification Technology* **2022**, 286, 120446.

8. J. Hu, T. Sun, X. Liu, Y. Guo, S. Wang, *RSC advances* **2016**, 6, 64039.

9. Q. Shi, J. Wang, H. Shang, H. Bai, Y. Zhao, J. Yang, J. Dong, J. Li, *Separation and Purification Technology* **2020**, 230, 115850.

10. S. Qadir, Y. Gu, S. Ali, D. Li, S. Zhao, S. Wang, H. Xu, S. Wang, *Chemical Engineering Journal* **2022**, 428, 131136.

11. X.-W. Liu, Y.-M. Gu, T.-J. Sun, Y. Guo, X.-L. Wei, S.-S. Zhao, S.-D. Wang, *Industrial & Engineering Chemistry Research* **2019**, 58, 20392.

12. Y. Chen, Y. Wang, Y. Wang, Q. Xiong, J. Yang, L. Li, J. Li, B. Mu, *AIChE Journal* **2022**, 68, e17819.

13. T. Li, X. Jia, H. Chen, Z. Chang, L. Li, Y. Wang, J. Li, *ACS Applied Materials & Interfaces* **2022**, 14, 15830.

14. C. E. Kivi, B. S. Gelfand, H. Dureckova, H. T. Ho, C. Ma, G. K. Shimizu, T. K. Woo, D. Song, *Chemical Communications* **2018**, 54, 14104.

15. X. Wu, B. Yuan, Z. Bao, S. Deng, *Journal of colloid and interface science* **2014**, 430, 78.

16. J. Liu, X. Tang, X. Liang, L. Wu, F. Zhang, Q. Shi, J. Yang, J. Dong, J. Li, *AIChE Journal* **2022**, 68, e17589.

17. Y. Guo, J. Hu, X. Liu, T. Sun, S. Zhao, S. Wang, *Chemical Engineering Journal* **2017**, 327, 564.

18. F. Zhang, H. Shang, B. Zhai, X. Li, Y. Zhang, X. Wang, J. Li, J. Yang, *AIChE Journal* **2023**, 69, e18079.

19. X. Ren, T. Sun, J. Hu, S. Wang, *Microporous and mesoporous materials* **2014**, 186, 137.

20. T.-H. Kim, S.-Y. Kim, T.-U. Yoon, M.-B. Kim, W. Park, H. H. Han, C.-i. Kong, C.-Y. Park, J.-H. Kim, Y.-S. Bae, *Chemical Engineering Journal* **2020**, 399, 125717.

21. M.-B. Kim, P. K. Thallapally, *Journal of Coordination Chemistry* **2021**, 74, 216.

22. U. Böhme, B. Barth, C. Paula, A. Kuhnt, W. Schwieger, A. Mundstock, J. r. Caro, M. Hartmann, *Langmuir* **2*013***, 29, 8592.

23. L. Yang, X. Cui, Q. Ding, Q. Wang, A. Jin, L. Ge, H. Xing, *ACS Applied Materials & Interfaces* **2019**, 12, 2525.

24. J. Pei, J.-X. Wang, K. Shao, Y. Yang, Y. Cui, H. Wu, W. Zhou, B. Li, G. Qian, *Journal of Materials Chemistry A* **2020**, 8, 3613.

25. M. Chang, J. Ren, Y. Wei, J.-X. Wang, Q. Yang, D. Liu, J.-F. Chen, *Separation and Purification Technology* **2021**, 279, 119656.

26. P. Zhang, L. Yang, X. Liu, J. Wang, X. Suo, L. Chen, X. Cui, H. Xing, *Nature communications* **2022**, 13, 4928.
